# Micro‐Strain Responsive Near‐Infrared Mechanoluminescence for Potential Nondestructive Artificial Joint Stress Imaging

**DOI:** 10.1002/adma.202505360

**Published:** 2025-09-08

**Authors:** Wenhao Li, Puxian Xiong, Xiaoxin Zheng, Luyue Niu, Lugui Cui, Qingyu Wang, Bruno Viana, Pieter Dorenbos, Jianzhong Zhang, Jing Ren

**Affiliations:** ^1^ Key Laboratory of In‐Fiber Integrated Optics of Ministry of Education, College of Physics and Optoelectronic Engineering Harbin Engineering University Harbin 150001 China; ^2^ Department of Electrical and Electronic Engineering The University of Hong Kong Hong Kong 999077 China; ^3^ Macau Institute of Materials Science and Engineering (MIMSE) Macau University of Science and Technology Taipa Macao 999078 China; ^4^ College of Nuclear Science and Technology Harbin Engineering University Harbin 150001 China; ^5^ PSL Research University Chimie Paris Tech IRCP CNRS Paris 75005 France; ^6^ Faculty of Applied Sciences, Department of Radiation Science and Technology Delft University of Technology Mekelweg 15 Delft 2629JB The Netherlands

**Keywords:** defect luminescence, micro strain response, near‐infrared mechanoluminescence, Sb^3+^, total knee replacement

## Abstract

Recently, joint replacement surgery is facing significant challenges of patient dissatisfaction and the need for revision procedures. In‐situ monitoring of stress stability at the site of artificial joint replacement during postoperative evaluation is important. Mechanoluminescence (ML), a novel “force to light” conversion technology, may be used to monitor such bio‐stress within tissues. However, this is hindered by ultraviolet–visible ML emission wavelength, low ML intensity, and high strain response sensitivity. Here, by incorporating Sb^3+^ ions into Sr_3_Sn_2_O_7_ crystals, a highly strain‐responsive material, with ML originating from intrinsic defect emissions is obtained. The Sr_3_Sn_1.98_Sb_0.02_O_6.99_ film produces detectable ML signals under compressive strain as low as 50 µst in the absence of biological tissue. After pre‐irradiating with red light through 15 mm of porcine tissue, ML signals can still be detected through the same tissue thickness. Notably, this material enabled real‐time stress imaging through 4 mm of porcine skin during mild finger joint bending. This work presents a novel methodological framework and proposes a new mechanism to defect ML. It offers a fresh perspective for designing high‐performance ML materials and lays the foundation for innovative research to enhance the functionality of artificial tissues and joints in living organism.

## Graphical abstract

In situ and real‐time stress sensing during total knee replacement procedure is expected to be realized based on near‐infrared mechanoluminescent phosphor of Sr_3_Sn_1.98_Sb_0.02_O_6.99_, which has the lowest strain sensitivity as low as 50 µst.

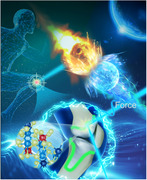

## 1 Introduction

Joint replacement surgery, practiced for over four decades and widely adopted worldwide, aims to improve the quality of life for individuals with knee arthritis.^[^ ^1^ ^]^ Its primary objectives encompass the mitigation of pain and the optimization of overall functional capacity. About 3 200 000 procedures were performed in the United States alone in 2023.^[^ ^2^ ^]^ However, implant placement marks only the beginning of postoperative care. Continuous monitoring is essential, as complications such as aseptic loosening, polyethylene wear, and infection can arise, leading to implant failure and the need for revision surgery. Notably, the revision rate for total knee replacement (TKR) is estimated up to 6%.^[^ ^3^ ^]^ These revision procedures are costly with irreversible joint damage, and in severe cases, may pose risks to the patient's life.^[^ ^4^ ^]^ The challenge of revision TKR remains a major clinical concern, significantly affecting patient satisfaction after surgery. Proper alignment of the joint and balanced soft tissue loading are critical for implant longevity and functional recovery.^[^ ^5^ ^]^ Clinical evidence suggests that the majority of TKR failures are associated with instability, wear, and loosening, and these are resulted from malalignment or excessive loading during daily activities.^[^ ^6^ ^]^ Thus, real‐time monitoring of biomechanical signals during routine movement can offer early warnings of adverse outcomes, enabling timely interventions such as manipulation under anesthesia and guiding personalized rehabilitation.^[^ ^5^, ^7^ ^]^ Continuous mechanical monitoring of joint implants is therefore essential for identifying abnormal stress distributions and preventing long‐term complications.

In recent years, smart orthopedic implants integrated with sensors have received growing attention for their ability to provide timely feedback on implant failure, inflammation, osteolysis or infection.^[^ ^8^ ^]^ Combining sensor data with active therapeutic interventions shows great potential in intelligent orthopedic care, such as instrumented joint prostheses equipped with strain gauges and wearable devices for joint motion monitoring.^[^ ^9^ ^]^ However, most conventional sensors detect mechanical stress by monitoring changes in electrical signals, such as resistance or capacitance. Other types of stress sensors, including piezoelectric, triboelectric, magnetic, and fiber Bragg grating sensors, are also explored.^[^ ^7^ ^]^ These methods, however, face significant challenges in the complex three‐dimensional sensing environment of biological tissues, where redundant interconnections and external devices complicate in situ stress monitoring and limit their clinical application.^[^ ^10^ ^]^ Recently, there has been a surge in novel stress sensors based on inorganic mechanoluminescent (ML) materials.^[^ ^11^ ^]^ These sensors leverage the linear relationship between ML intensity and applied stress, enabling accurate stress quantification. Due to their unique luminescent mechanisms, stable physicochemical properties and excellent compatibility, the developed ML sensors exhibit remarkable reproducibility, passive operation, miniaturization potential, non‐toxicity, and the ability to visualize stress information. However, the utility of ML signals in the ultraviolet‐visible (UV‐Vis) range poses a challenge for in vivo stress detection, as their emission can poorly penetrate through biological tissues, hindering the achievement of in situ, real‐time monitoring. In contrast, near‐infrared (NIR) ML materials demonstrate superior tissue penetration and spatial resolution, making them particularly advantageous for biological stress sensing applications.^[^ ^12^ ^]^

Consequently, researchers have made significant progress in developing high‐performance NIR ML materials, utilizing luminescent centers such as Nd^3+^, Er^3+^, Yb^3+^ and Cr^3+^ in the deep‐red to NIR spectral range. ^12^, ^13^ ^]^ These advancements have demonstrated potential applications in in vivo stress/strain distribution and dynamic optical imaging. Nevertheless, challenges remain in achieving high‐performance NIR ML materials, including weak luminescence intensity, limited charging wavelength (usually UV lights), inability to detect low strain signals (below 200 µst), and unresolved uncertainties regarding the impact of external forces and defect types on ML behavior.^[^ ^14^ ^]^ Currently, there is no consensus on the theoretical underpinnings of ML origins and its complex stress response mechanisms, which highlights the urgent need for convincing ML mechanism models. Additionally, many existed NIR ML materials are limited to force measurements in the tens to hundreds of newtons, inadequate for applications such as artificial joints that may experience loads exceeding 1000 N. Thus, the development of novel rigid matrix ML materials for in situ stress detection in biological joints is essential.

**Figure 1:**
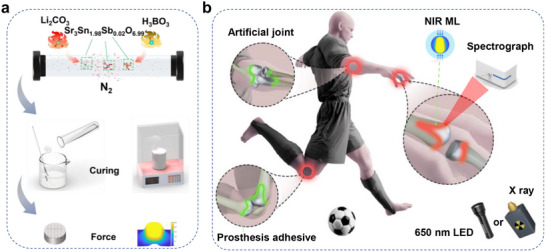
a) Schematic diagram of ML powder and corresponding ML test sample preparation process. The ML powder is synthesized by sintering a mixture of crystalline matrix precursors, flux, and dopants in an oxygen‐deficient N_2_ atmosphere. Subsequently, the ML powder is mixed with epoxy resin to fabricate ML composite samples, which exhibit clear ML under applied stress. b) Schematic diagram of potential in situ stress detection in joint replacement surgery. The ML sample is pre‐irradiated externally with 650 nm light or X‐rays, after which NIR ML signals can be detected through biological tissue. This enables non‐destructive in vitro stress detection at the joint replacement site using a NIR spectrometer.

In this study, we introduced a novel broadband NIR ML material Sr_3_Sn_1.98_Sb_0.02_O_6.99_, and demonstrated through a series of comparative experiments that its ML may originate from defects. First, it is widely known that existed Sb‐based ML or photoluminescent phosphors typically exhibit emission peaks at ≈460–562 and 620–810 nm, arising from ns^2^‐type ^1^P_1_‐^1^S_0_ and ^3^P*_n_*‐^1^S_0_ transitions (where *n* can be 0, 1, 2). For example, Li et al. developed Sb‐doped CaZnOS‐based materials for ML, with emission peaks in both ML and photoluminescence spectra peaked at 465 and 620 nm.^[^ ^15^ ^]^ In contrast, Sb‐doped photoluminescent materials have been more widely studied, such as the work by Su et al., who developed a luminescent material of Sb^3+^ doped Cs_2_ZnCl_4_, with photoluminescent peaks at 562, 745 and 810 nm.^[^ ^16^ ^]^ These materials primarily use Sb^3+^ as a luminescent center, but not mention the defect regulation work based on this ion. In contrast, Sr_3_Sn_1.98_Sb_0.02_O_6.99_ exhibits a single broadband emission peaked ≈800 nm. Generally, the emission of Sb^3+^ will redshift along the nephelauxetic sequence, but a shift toward 800 nm in the stannate seems quite large.^[^ ^17^ ^]^ Notably, a similar emission peak at 800 nm is observed even in pristine Sr_3_Sn_2_O_7_, highlighting its unique properties compared to previously reported Sb‐activated luminescent materials.^[^ ^15^, ^16^, ^18^ ^]^ Compared with classical ML materials such as Sr_3_Sn_2_O_7_ (A2_1_am): Nd^3+^, CaZnOS: Nd^3+^, and LiNdO_3_: Nd^3+^, this NIR ML material achieves exceptional performance under stress ranging from 200 to 1400 N.^[^ ^13^, ^14^, ^19^ ^]^ Furthermore, when fabricated into an “ML ring”, the material enabled the detection of distinct in situ ML signals through 4 mm of porcine skin during slight finger joint bending. In biosensing experiment, the material was successfully pre‐irradiated through 15 mm of porcine tissue using 650 nm red light and produced a clear ML signal detectable through the same tissue thickness. Notably, the defect‐mediated luminescence of this pristine ML material allows for a more direct investigation of the relationship between defects and ML, devoid of electronic transfer processes to ionic luminescent centers. We elucidated the NIR ML mechanism through first‐principles calculations and experimental characterizations. By introducing multiple defects and engineering the energy band structure, we lowered the electronic transition barrier toward enhanced defect luminescence. Remarkably, the Sr_3_Sn_1.98_Sb_0.02_O_6.99_ film demonstrated the lowest strain detection threshold among reported NIR ML materials. This study offers new perspectives for the development of novel ML materials. High ML performance is observed and we propose a defect‐mediated luminescence model, paving the way for the advancement of high‐performance ML materials (**Figure** **1**).

## 2 Results and Discussion

### 2.1 Crystal Phase and Microstructure

**Figure 2:**
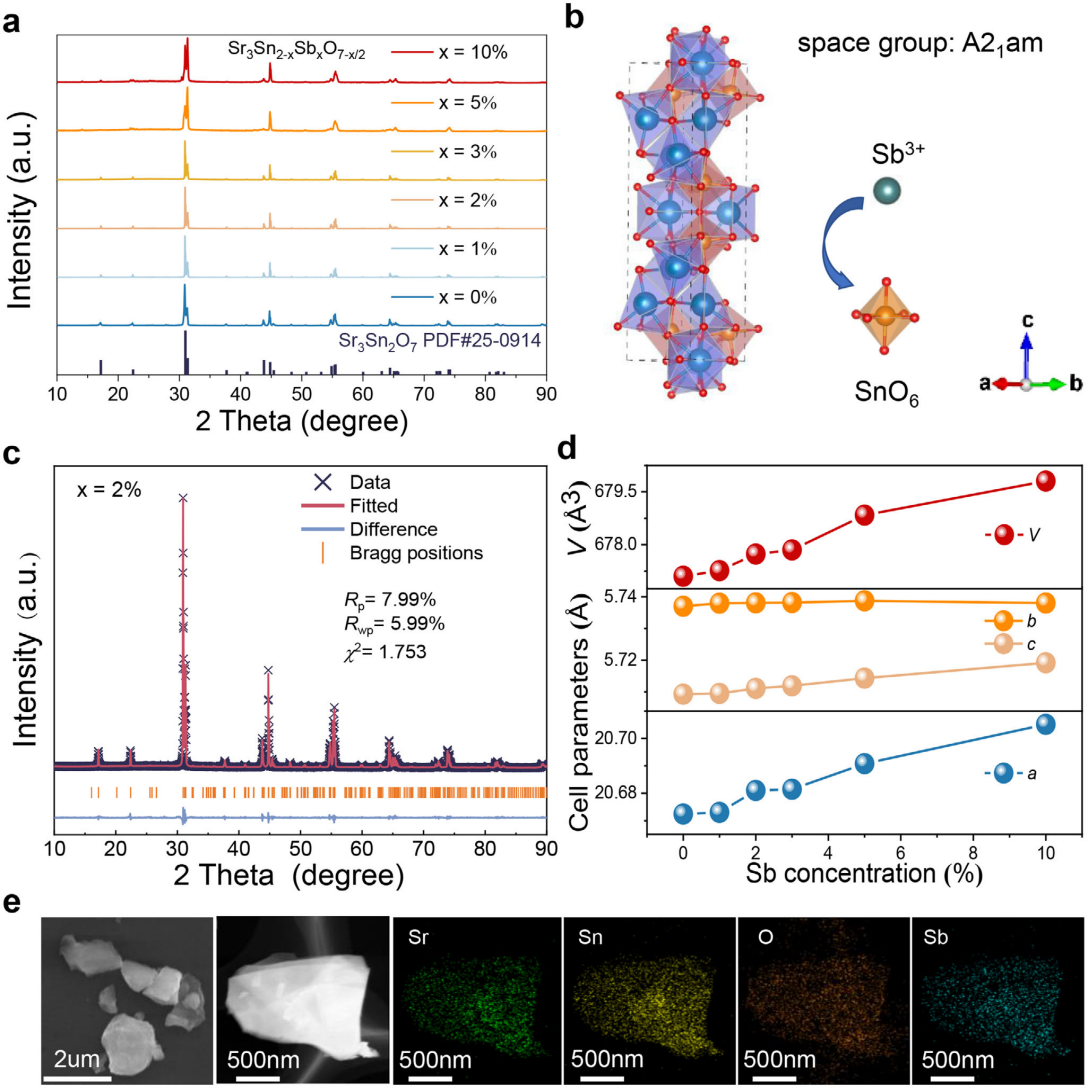
a) XRD patterns of Sr_3_Sn_2‐x_Sb_x_O_7‐x/2_ (*x* = 0, 1, 2, 3, 5, 10 mol.%). The standard card of Sr_3_Sn_2_O_7_ (PDF #25‐0914) is provided at the bottom. b) Crystal structure of Sr_3_Sn_2_O_7_ illustrating the coordination state of Sr^2+^ and Sn^4+^, and the substitution of Sn^4+^ by Sb^3+^. c) Rietveld refinement of the Sr_3_Sn_1.98_Sb_0.02_O_6.99_. The black crosses and red solid lines represent the experimental and calculated patterns, respectively. Vertical bars indicate the Bragg peak positions, and the blue line at the bottom shows the residuals. d) lattice parameters (a, b, c, and V represent the unit cell dimensions along the x‐, y‐, and z‐axes, and the unit cell volume, respectively) obtained from Rietveld refinement at different Sb^3+^ doping concentrations. e) SEM image, elemental mapping images of Sr_3_Sn_1.98_Sb_0.02_O_6.99_.

X‐ray diffraction (XRD) patterns for the samples with different doping concentrations (*x* = 0, 1, 2, 3, 5, 10 mol.%) of Sr_3_Sn_2‐x_Sb_x_O_7‐x/2_ are depicted in **Figure** **2**a. Comparison with the reference XRD pattern of Sr_3_Sn_2_O_7_ confirms that all the samples retain the characteristic orthorhombic Sr_3_Sn_2_O_7_ (PDF #25‐0914), indicating that the incorporation of Sb^3+^ does not induce any significant phase changes. The crystal space group of the synthesized Sr_3_Sn_2_O_7_ is determined to be A2_1_am,^[^ ^14^, ^20^ ^]^ as illustrated in Figure 2b. The XRD Rietveld refinement of Sr_3_Sn_1.98_Sb_0.02_O_6.99_ is shown in Figure 2c, while the refinement results for samples with other Sb doping concentrations are provided in Figure S2 (Supporting Information). The experimental pattern matched well with the calculated pattern. As shown in Figure 2d, the crystal volume (*V*) and lattice parameters *a* and *c* increase with the Sb doping concentration, while the lattice parameter *b* shows no significant variation. The increase in crystal volume confirms the successful incorporation of Sb^3+^ ions into the Sr_3_Sn_2_O_7_ lattice. Scanning electron microscopy (SEM) images reveal a fragmented structure of the synthesized material with an average particle size of ≈2 µm (Figure 2e). Energy‐dispersive X‐ray spectroscopy (EDS) elemental mapping shows the presence and homogeneous distribution of Sr, Sn, O and Sb within the particle.

### 2.2 PL Properties of Sr_3_Sn_2‐x_Sb_x_O_7‐x/2_

Notably, the positions of the PL excitation peak with different Sb^3+^ doped samples do not change significantly (Figure S3a, Supporting Information). Under 254 nm excitation, Sr_3_Sn_2‐x_Sb_x_O_7‐x/2_ (*x* = 0.0–10.0%) exhibit broadband NIR emission (700–1000 nm, FWHM > 100 nm) (Figure S3b, Supporting Information). The strongest PL intensity is observed when *x* = 1%, and as *x* increases further, the PL intensity gradually decreases. Room‐temperature luminescence decay curves (λ_ex_ = 254 nm, λ_em_ = 800 nm) were measured (Figure S3c,d, Supporting Information). The measured lifetime is in the order of several hundred µs, significantly longer than the typical lifetime of Sb^3+^‐based luminescent materials, which are usually in the tens of µs.^[^ ^15^, ^16^ ^]^ As Sb^3+^ concentration increases, PL lifetime decreases from 807 to 429 µs.

### 2.3 ML Properties of Sr_3_Sn_2‐x_Sb_x_O_7‐x/2_

**Figure 3:**
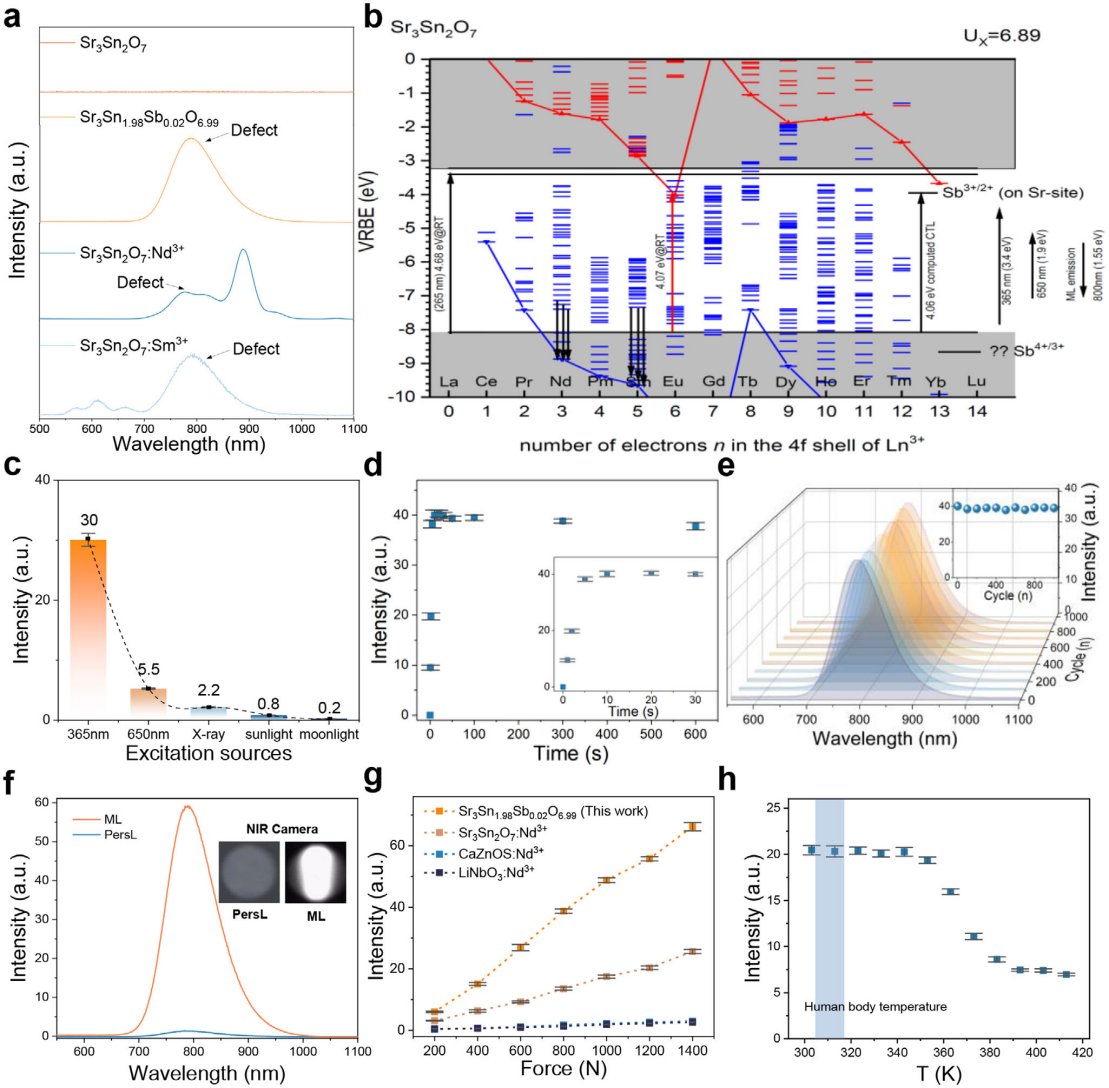
a) ML spectra of Sr_3_Sn_2_O_7_, Sr_3_Sn_1.98_Sb_0.02_O_6.99_, Sr_3_Sn_2_O_7_:Nd^3+^ and Sr_3_Sn_2_O_7_:Sm^3+^ under a 1000 N compressive force. Pre‐irradiated by 365 nm UV LED (Light‐Emitting Diode) for 1 min, tested after 1 min in dark environment. b) VRBE diagram of Sr_3_Sn_2_O_7_ showing the ground‐state energy levels of lanthanide ions and Sb ion. Arrows indicate experimentally observed transitions. c) ML spectra of Sr_3_Sn_1.98_Sb_0.02_O_6.99_ under a 1000 N compressive force, following 1 min pre‐irradiation by different light sources. d) ML intensity of Sr_3_Sn_1.98_Sb_0.02_O_6.99_ under a 1000 N compressive force as a function of UV irradiation time. Inset: enlarged view of the 0 to 30 s irradiation interval. e) ML spectra of Sr_3_Sn_1.98_Sb_0.02_O_6.99_ under 1000 cyclic compressive forces loadings. Inset: variation of ML peak intensity. f) ML and PersL spectra of Sr_3_Sn_1.98_Sb_0.02_O_6.99_. The insets show in situ luminescence images captured by a NIR camera. g) Comparison of the ML response of Sr_3_Sn_1.98_Sb_0.02_O_6.99_ under varying stress levels after UV charging with that of Sr_3_Sn_2_O_7_ (A2_1_am): Nd^3+^, CaZnOS: Nd^3+^ and LiNdO_3_: Nd^3+^. h) ML intensity of Sr_3_Sn_1.98_Sb_0.02_O_6.99_ under a 1000 N compressive force measured at various temperatures.

The emission band ranging from 650 to 1000 nm originates from defect energy levels within the host lattice of Sr_3_Sn_2‐x_Sb_x_O_7‐x/2_. To verify the origin of this emission, we synthesized pristine Sr_3_Sn_2_O_7_ as well as Sb^3+^, Nd^3+^ and Sm^3+^ doped Sr_3_Sn_2_O_7_, and measured their ML spectra under identical conditions (**Figure** **3**a). The XRD patterns of these samples confirm that they all retain the orthorhombic Sr_3_Sn_2_O_7_ (PDF #25‐0914) phases (Figure S6, Supporting Information). As shown in Figure 3a, pure Sr_3_Sn_2_O_7_ exhibits no ML signal. Upon Sb doping, an obvious broadband NIR emission emerges (peaked ≈800 nm). In contrast, Sr_3_Sn_2_O_7_:Nd^3+^ shows multiple overlapping emission peaks in 650–1100 nm. Peak fitting analysis identifies six emission peaks at 766, 797, 830, 888, 945 and 1067 nm (Figure S7, Supporting Information). These peaks correspond to transitions of Nd^3+^ ions and the defect emission, respectively. The defect emission peak is more pronounced in Sr_3_Sn_2_O_7_:Sm^3+^ (Figure 3a). The ML emission consists of defect‐related 800 nm and Sm^3+^‐related emissions at 568, 612, and 665 nm, corresponding to the Sm^3+ 4^G_5/2_ → ^6^H_5/2_, ^4^G_5/2_ → ^6^H_7/2_, and ^4^G_5/2_ → ^6^H_9/2_ transitions, respectively (Figure S7, Supporting Information). To compare the energy relationships between the conduction band (CB), valence band (VB), rare‐earth ion electronic states, and defects in the host crystal, we constructed a vacuum‐referenced binding energy (VRBE) diagram based on the chemical shift model and spectroscopic data of rare‐earth ions.^[^ ^21^ ^]^ Here, the VB to Eu^3+^ charge transfer (CT) energy is 4.07 eV, and the energy for host exciton creation is 4.68 eV.^[^ ^22^ ^]^ Using these values, we developed the VRBE scheme shown in Figure 3b.^[^ ^23^ ^]^ The diagram reveals that the Sm^4+/3+^ and Nd^4+/3+^ CT levels are located within the VB. We have tentatively placed the Sb^4+/3+^ CT level inside the VB near −8.5 eV. The ML mechanism is further illustrated in Figure 3b and Figure S8 (Supporting Information). After pre‐irradiation by UV light excitation, defect centers capture and store electrons. When the excitation light is turned off, an induced electric field generated under stress excites the stored electrons to the CB. These electrons are subsequently captured by defect and ion luminescence centers. Additionally, energy transfer occurs between luminescent defects and ion luminescence centers, accompanied by energy loss (e.g., through non‐radiative relaxation).^[^ ^24^ ^]^

In Sr_3_Sn_2_O_7_: Nd^3+^, energy from defect luminescence (800 nm, 1.5 eV) is transferred to the Nd^3+^ ion luminescence center (887 nm, 1.4 eV). During this process, significant energy loss occurs, leading to a much lower ML intensity compared to the direct defect‐driven emission observed in Sr_3_Sn_1.98_Sb_0.02_O_6.99_ (Figure 3g). In Sr_3_Sn_2_O_7_: Sm^3+^, energy from the Sm^3+^ ion luminescence center (568 nm, 2.18 eV) is transferred to the defect luminescence center (800 nm, 1.5 eV). These mechanisms are consistent with the observed spectral peak intensity trends: in Sr_3_Sn_2_O_7_: Nd^3+^, the luminescence intensity of the Nd^3+^ ion center (887 nm) is significantly stronger than that of the defect luminescence (800 nm). In Sr_3_Sn_2_O_7_: Sm^3+^, the intensity of the defect luminescence (800 nm) is significantly stronger than that of the Sm^3+^ ion luminescence center (568 nm). These results reveal that single‐doped samples (Sb^3+^, Nd^3+^, Sm^3+^) all exhibit a prominent emission band ≈800 nm, whose intensity is closely related to the energy transfer mechanisms of defect levels. This indicates that the NIR emission at 800 nm originates from direct defect‐driven luminescence, rather than from ionic luminescent centers.

For optimal ML sensing performance, the Sr_3_Sn_1.98_Sb_0.02_O_6.99_ sample was selected for subsequent experiments. We evaluated the charging efficiency of different excitation sources on the ML sample by 365 nm UV light, 650 nm red light, X‐rays (total dose of 720 mGy), sunlight, and moonlight in Figure 3c. (The tests were conducted on July 12, 2024, with measurements taken during the day from 11:00 AM to 12:00 PM and at night from 11:00 PM to 12:00 AM. The location coordinates were 126°40′55″E longitude and 45°46′19″N latitude). The ML testing setup is illustrated in Figure S9a (Supporting Information). Clearly, 365 nm UV light produces the strongest ML intensity, followed by 650 nm red light, with an intensity ≈2/11 that of UV light excitation. Given the superior tissue penetration of 650 nm red light compared to UV light, red‐light charging holds significant potential for non‐invasive stress detection within biological tissues.

Additionally, ML intensity under low‐dose‐rate X‐ray excitation (total radiation dose of 720 mGy) was about half that observed with 650 nm red light. As the X‐ray dose rate increased, the ML intensity is significantly improved (Figure S10, Supporting Information). However, excessive X‐ray power poses challenges for in vivo applications. The sample also exhibited detectable ML signals under sunlight and moonlight charging (Figure S11, Supporting Information). Most reported ML materials require a charging time of 1 to 2 min. In contrast, our material achieves significantly faster charging efficiency in Figure 3d. After just 5 s of UV irradiation, the ML intensity reaches a high level, and further irradiation up to 600 s does not result in a noticeable intensity increase. This indicates that the sample achieves full charging within only 5 s. The behavior of ML intensity during cyclic force loading and UV‐recharging is depicted in Figure 3e, revealing the recovery of ML to the initial values each time after recharging. As shown in the inset, after 1000 compression cycles, the ML intensity remains remarkably stable. In contrast, after a single pre‐irradiate process, the ML intensity decreases significantly with repeated compressions. Its ML intensity decayed gradually with the increase of cycle number and tends to be stable after the 50th cycle. We confirmed that the ML intensity remains detectable after 100 cycles. (Figure S12, Supporting Information). This behavior may be attributed to the continuous energy transfer from deep defect energy levels ≈1.08 eV to shallower levels ≈0.79 eV (Figure S13a,d, Supporting Information). Additionally, in most ML materials, strong persistent luminescence (PersL) often complicates the analysis of corresponding ML responses.^[^ ^25^ ^]^ However, here, testing the PersL and ML signals 1 min after charging shows that the ML intensity is at least 45 times stronger than the PersL intensity shown in Figure 3f. ML response of Sr_3_Sn_1.98_Sb_0.02_O_6.99_ is compared with previously reported Sr_3_Sn_2_O_7_ (A2_1_am): Nd, CaZnOS: Nd^3+^ and LiNdO_3_: Nd^3+^ in Figure 3g. All samples exhibited good linearity within the wide pressure range of 200–1400 N (Table S3, Supporting Information). The strong linear fit (*R* ^2^ > 0.99) underscores the potential of this phosphor for highly sensitive force‐sensing applications. Sr_3_Sn_1.98_Sb_0.02_O_6.99_ exhibits the strongest ML signal, with an integrated intensity under 1400 N pressure in the 600 – 1000 nm range. It is ≈3.6 times that of Sr_3_Sn_2_O_7_ (A2_1_am): Nd^3+^, 150 times that of CaZnOS: Nd^3+^, and 600 times that of LiNdO_3_: Nd^3+^ phosphors.

From application perspective, it is crucial to distinguish the potential mutual influence of force and temperature on the recorded luminescence signals. ML spectra at various temperatures were also collected, with the inset highlighting the biological temperature range in 307–318 K, (Figure 3h; Figure S9b, Supporting Information). The ML intensity remains stable between 303–353 K. However, as the temperature increases further, a significant decrease in ML intensity is observed in the 353 to 393 K range. Beyond this, the ML intensity gradually decreases with continued temperature elevation. Due to the degradation of the mechanical properties of the organic epoxy resin matrix at temperature above 413 K, further temperature increases were not pursued. The observed significant variations in ML intensity with temperature can be predicted by thermoluminescence (TL) glow curves. As shown in Figure S13a,d (Supporting Information), three main TL peaks are observed at 360, 397, and 539 K, which are labelled as Peak 1, Peak 2, and Peak 3, respectively. Below 353 K, the depths of these defect levels are too deep to be significantly affected by temperature changes. However, as the temperature approaches 360 K, Peak 1 related defect carriers are released, leading to a sharp drop in ML intensity. Then at ≈393 K, carriers from Peak 2 are released, further reducing the ML intensity. Peak 3 is deep enough so that further temperature increases cannot release such trapped carriers. This results in clear ML signal until the upper working limit of the organic matrix (413 K).

### 2.4 Defect Related Theoretical Calculation Prediction

To further elucidate the nature of ML‐active defects such as oxygen vacancies, we employed first‐principles calculations to predict the type of defects stabilized in Sr_3_Sn_1.98_Sb_0.02_O_6.99_, and their distribution within the bandgap. In most ML materials, the ML performance is intricately related to the efficient transfer and recombination of charge carriers between the host material and ionic luminescent centers. The presence of appropriate defects enhances this process by facilitating the storage and transfer of charge carriers. Under applied stress, such carriers are released and transfer energy to the ionic luminescent centers to produce ML. Conversely, defect‐emitting ML materials exhibit direct emission from crystal defects, eliminating the need for electron transfer to ionic luminescent centers. This allows for a more straightforward investigation of the relationship between defects and ML. Research on ML in defect‐emitting materials contributes to a deeper understanding of the mechanisms associated with defects and trap states, thereby advancing the theoretical design of ML materials.

**Figure 4:**
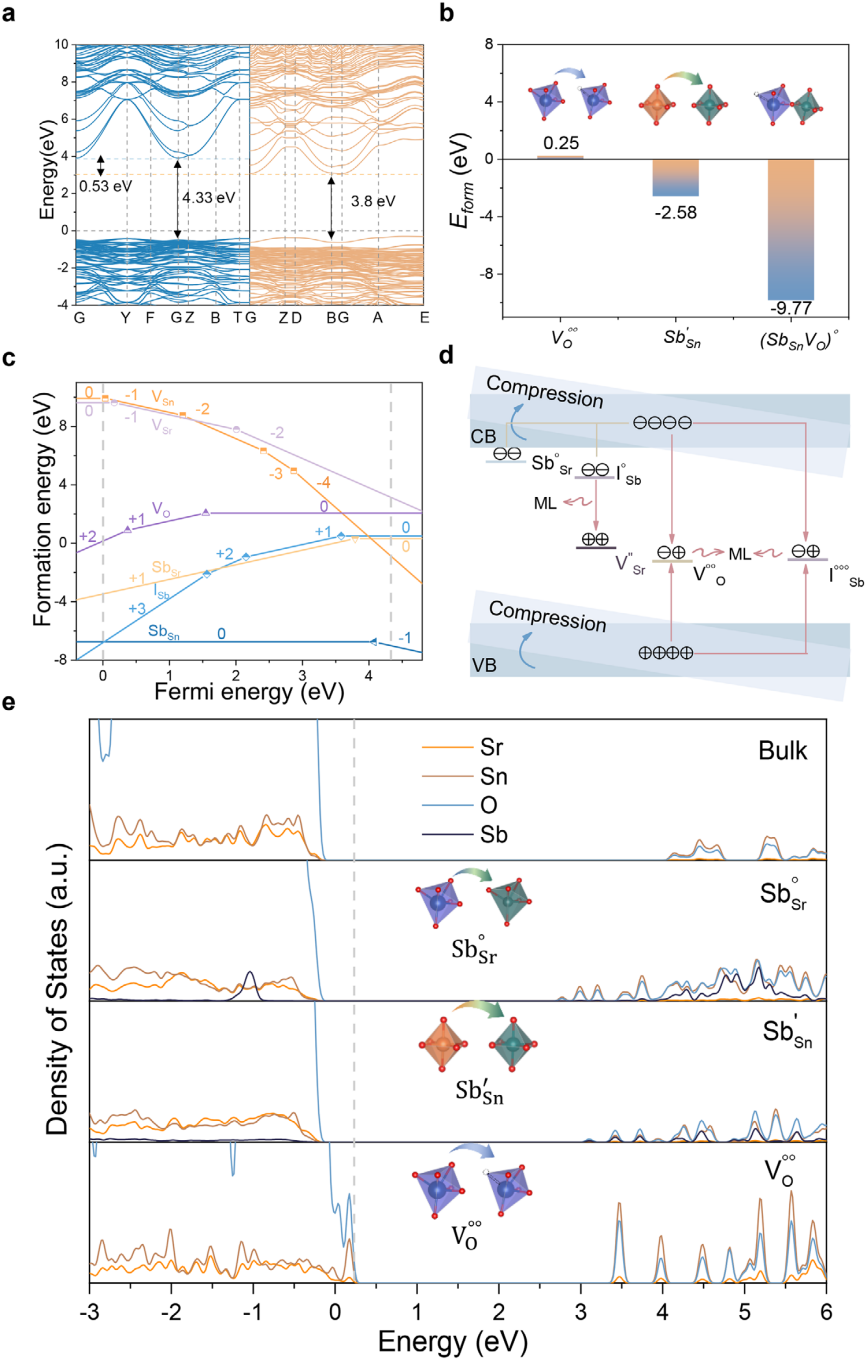
a) Calculated band structure of Sr_3_Sn_2_O_7_ and Sr_3_Sn_2_O_7_:Sb. The conduction band minimum (CBM) is shown by dashed lines, and the bandgap values are marked with arrows. b) Formation energies (*E_form_*) of various defects in the Sr_3_Sn_2_O_7_. The inset shows a schematic illustration of the defect structure. c) Charged defect formation energies of vacancy, substitute and interstitial defects in Sr_3_Sn_2_O_7_. d) Thermodynamic transition levels of vacancy, substitute and interstitial defects. Values below the lines indicate the energy positions relative to the VBM. e) Partial density of states for the stable charge states of *Sb_Sr_*, *Sb_Sn_*, and V_O_ defect and defect‐free Sr_3_Sn_2_O_7_. The insets are schematic drawings of defective structures.

To explore this further, we employed density functional theory (DFT) to compute the band structure of pristine and defect‐containing states, alongside various intrinsic and dopant‐induced defects/traps. For the optimized orthorhombic structure of Sr_3_Sn_2_O_7_ and the Sb‐doped Sr_3_Sn_1.98_Sb_0.02_O_6.99_, the estimated band gaps are 4.33 and 3.8 eV, respectively (**Figure** **4**a). The introduction of Sb into the crystal significantly reduces the band gap, attributed to the bonding interactions between Sb and O^2−^ neighboring ions. This doping also disrupts the lattice symmetry of the host material, leading to lattice distortion and subsequent alterations in the band structure. To identify the most likely type of defect, we calculated the defect formation energies (*E_form_*) for a series of defects in Sr_3_Sn_1.98_Sb_0.02_O_6.99_ (Figure S16, Supporting Information). Lower formation energies suggest that less energy is required for defect formation, making such defects more likely to be formed. Notably, negative *E_form_* values indicate that defects can form spontaneously. The results show that $Sb_{Sr}^{\circ }$, $Sb_{Sn}^{\prime }$, $I_{Sb}^{\circ \circ \circ }$ and ${\left(Sb_{Sn}V_{O}\right)}^{\circ }$ have negative formation energies, suggesting they are most likely to exist, whereas $V_{Sr}^{\prime \prime }$, $V_{Sn}^{\prime \prime \prime \prime }$, and $V_{O}^{\circ \circ }$ have positive formation energies, requiring external energy to form. Interestingly, as shown in Figure 4b, the formation energy of $V_{O}^{\circ \circ }$ decreases significantly with the substitution of Sb for Sn, indicating that this substitution facilitates the generation of oxygen vacancies.

To further investigate the role of defects in ML and their potential mechanisms, we examined the defects of *V_Sr_*, *V_Sn_*, *V_O_*, *Sb_Sr_*, *I_Sb_*, and *Sb_Sn_*, revealing their relationships with the Fermi level (E_F_) across various charge states (as shown in Figure 4c). The chemical potential range utilized in the calculations is illustrated in Figure S16 (Supporting Information), with lines DE and FG corresponding to the SrO_2_ and SnO_2_ reference states, respectively. The blue‐shaded region enclosed by points D, E, G, and F delineates the thermodynamically allowed range of chemical potentials. Transition points are marked by shifts in slope, indicating the presence of thermodynamic transition levels. For example, ɛ(q_1_/q_2_) denotes the points at which the Fermi energies of charge states q_1_ and q_2_ align. The roles and impacts of intrinsic defects such as *V_Sr_*, *V_Sn_*, and *V_O_*, along with extrinsic defects like *Sb_Sr_*, *I_Sb_*, and *Sb_Sn_*, are illustrated in Figure 4c, where these defects generate a rich array of defect transition energy levels within the band gap. Notably, the +1, +2, and +3 valence states of Sb at interstitial sites, as well as the +1 state of Sb substituting for Sr and the ‐1 state for Sn, exhibit negative defect formation energies. This suggests a tendency for easier formation under identical conditions and a propensity for spontaneous formation in energetically stable configurations. Conversely, the creation of Sr, Sn, and O vacancies, along with the 0‐valence state of Sb substituting for Sr, requires external energy. This observation is consistent with the high ambient temperature of 1550 °C, employed under a pure nitrogen atmosphere during the preparation process.

Dynamic transition energy levels were calculated for charge states associated with vacancy, substitution, and interstitial defects (Figure 4d). These defect levels function as acceptors, capturing electrons from the CBM or donor levels. The resulting energy storage and release between the defect levels, CB, and VB facilitate the generation of ML. Based on the valence band maximum (VBM), the transition level ɛ(+1/0) for *I_Sb1_* and ɛ(+1/0) for *Sb_Sr1_* are ≈3.58 and 4.06 eV, respectively. Both levels are situated close to the CB, indicating that the stored electrons can easily be released into the CB under stress contributing to ML. Hence, they may act as deep donors. *V_Sr_* and *V_Sn_* exhibit five transition levels within the band gap: ɛ(0/−1) of *V_Sr1_*, ɛ(−1/−2) of *V_Sr2_*, ɛ(0/−1) of *V_Sn1_*, ɛ(−1/−2) of *V_Sn2_*, and ɛ(−2/−3) of *V_Sn3_*, located at 0.18, 2.01, 0.04, 1.20, and 2.42 eV above the VBM, respectively. Considering the possible origin of ML, the energy gap between *I_Sb1_* and *V_Sr2_* is 1.57 eV, suggesting that the acceptor levels may be formed by *V_Sr2_* and *V_Sn3_*. Additionally, the transition levels ɛ(+2/+1) of *I_Sb2_*, ɛ(+3/+2) of *I_Sb3_*, ɛ(+1/0) of *V_O1_*, and ɛ(+2/+1) of *V_O2_* are located at 2.15, 1.56, 1.54, and 0.37 eV, respectively. The energy gaps between *I_Sb3_* and the VB, and between *V_O1_* and the VB, are ≈1.56 and 1.54 eV, respectively. This indicates that *I_Sb3_* and *V_O1_* may serve as donor levels, with electrons recombining with holes in the VB to generate ML emissions. Notably, the luminescence in this ML material arises directly from electron transitions associated with crystalline defects, bypassing the need for electron transfer processes from defects to ionic luminescent centers. The reduced energy transfer steps contribute to a higher efficiency of energy transfer, which is a key reason for the significantly enhanced luminescence intensity observed in this material.

The character of defect states is influenced by atomic relaxation and the electronic structures specific to the most stable charge states of dominant defects.^[^ ^26^ ^]^ We calculated the partial density of states for the most stable charge state structures of various defect systems (Figure 4e). The structures of Sb substituted Sr and Sn are stable in the +1 and −1 charge states, respectively, with *Sb_Sr_* acting as a donor defect and *Sb_Sn_* as an acceptor defect. Both defects generate a rich array of defect states near the CBM, primarily composed of O 2p, O 2s, Sn 5s, and weak contributions from Sr 5s and Sb 5s orbitals (Figure S17, Supporting Information). Although the contribution of Sb ions to the intensity of newly formed defect states is limited, Sb ions influence orbital hybridization by substituting for cation lattice sites of Sr and Sn, resulting in the formation of shallow‐level defect states. In contrast to cation substitutions, when oxygen vacancies are created, the oxygen vacancy remains stable in the +2 charge state as a donor, generating defect states located at 0.04 and 0.17 eV above the VBM. These defect states mainly correspond to O 2p, Sn 5s, and Sn 5p orbitals. The presence of these defect states significantly reduces the band gap, specifically, the substitutional defects *Sb_Sr_* and *Sb_Sn_* lower the band gap by generating new defect states at the CBM, while the vacancy defect *V_O_* creates states at both the VBM and CBM, further contributing to the energy bandgap reduction. This decrease in band gap energy lowers the energy barrier for electrons in the defects to transition to the CB, facilitating energy transfer between the host material and the luminescence center. The energy bandgap reduction is a critical factor for the significant enhancement of ML in the material, allowing the evolution from non‐emissive to emissive behavior.

### 2.5 Microstrain Performance and Potential Bio Applications

Here, upon UV excitation, the ML samples produced clear in situ emissions under finger pressure (Video S1, Supporting Information). This finding suggests that Sr_3_Sn_1.98_Sb_0.02_O_6.99_ has potential microstrain sensing ability. Given the importance of assessing the mechanical behavior at the bone–implant interface, this study may serve as a foundational step in bone biomechanics. It offers valuable insight into stress distribution and mechanical performance in bone and implants, and may help identify failure mechanisms under complex loading conditions. Real‐time biomechanical data also support personalized rehabilitation plans and allow patients to engage in optimally controlled exercise, reducing the risk of implant failure due to underuse or overexertion. Clinically, smart knee prostheses with strain, stress and gait monitoring ability have been shown to detect biomechanical signals during daily activity, enabling early warnings of postoperative complications and guiding timely interventions, such as manipulation under anesthesia.^[^ ^7^, ^9^ ^]^ Most existed bone stress sensors are based on resistive or piezoelectric effects, typically requiring complex circuitry and large device sizes, which limit their use for in vivo, in situ stress monitoring.^[^ ^7^ ^]^ In contrast, ML materials offer significant advantages, including small size, battery‐free operation, and compatibility with prosthesis adhesives, making them highly promising for real‐time stress sensing in biological environments. For example, ML powders are synthesized via a high‐temperature solid‐state reduction method, offering a simple route amenable to large‐scale production. Their small size and excellent stability further facilitate large‐area processing and integration. To explore the material's potential for in vivo strain detection, we conducted microstrain sensing and biomechanical sensing tests. Before any biological applications, it is crucial to assess the cytotoxicity of the sample. To this end, we conducted a thorough cell viability assay involving three distinct cell lines: mouse epithelioid fibroblasts (L929), human umbilical vein endothelial cells (HUVEC), and mouse embryonic osteoblast precursor cells (MC3T3‐E1). These cell lines were subjected to a range of concentrations of solutions containing powdered samples (Figure S18, Supporting Information). Biocompatibility was assessed across concentrations from 0 µg mL^−1^ (control) to 1000 µg mL^−1^. Cell viability remained above 90% across all samples. No significant differences were observed between the experimental groups and the negative control (all adjusted *P* > 0.82), indicating that the material exhibited no detectable cytotoxicity within the tested concentration range.

**Figure 5:**
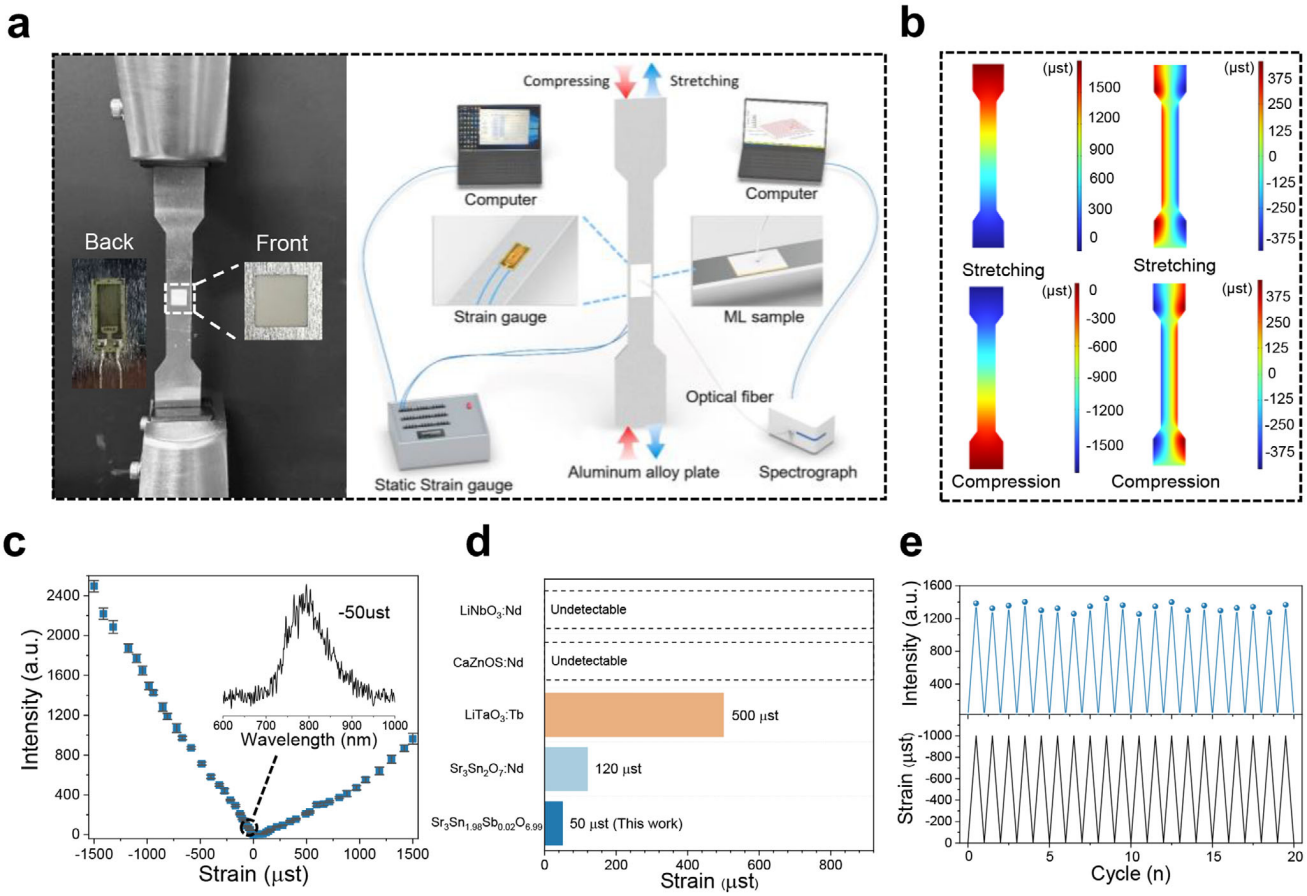
a) Schematic diagram of the tensile strain test. b) Simulation of longitudinal and transverse strains during the stretching and compression of the sample. c) ML intensity of the Sr_3_Sn_1.98_Sb_0.02_O_6.99_ sample under varying levels of applied microstrain. ML sample energized with 365 nm UV for 1 min, tested after 1 min in dark. The inset shows the ML spectrum at 50 µst. d) Comparison of the minimum absolute microstrain sensitivity reported and that of typical NIR ML materials. e) Strain and ML intensity variation of the Sr_3_Sn_1.98_Sb_0.02_O_6.99_ sample over 20 consecutive deformation cycles.

The schematic diagram for the tensile and compressive strain tests is illustrated in **Figure** **5**a, where a regularly shaped ML film and strain gauge sensor were meticulously adhered to the same locations on both sides of an aluminum alloy sheet. The inset in the figure provides an enlarged view of the actual sample. We employed a spectrometer and dynamic strain tester simultaneously for spectral and strain measurements. Additionally, to analyze the impact of different strain modes on the ML performance of the sample during tensile and compressive testing of the aluminum alloy sheet, we simulated the strain distribution throughout the deformation process using COMSOL software. As shown in Figure 5b, the aluminum alloy sample exhibited longitudinal tensile strain accompanied by transverse compressive strain, and conversely, during longitudinal compressive strain, it experienced transverse tensile strain. The relationship between the longitudinal and transverse strain components adheres to the elastic deformation constant, known as Poisson's ratio (≈0.3):

(1) $$v=\left.-\varepsilon \right|/\varepsilon$$

where ɛ| represents the strain perpendicular to the load direction and ɛ denotes the strain in the load direction. Figure 5c illustrates that the sensitivity of the ML film under longitudinal compressive strain is significantly lower than that under longitudinal tensile strain. A microstrain of 50 µst can elicit a discernible ML signal under compressive strain (in the inset of Figure 5c), while a threshold of 160 µst is required to detect a signal under tensile strain. The sensitivity threshold for tensile strain is ≈0.32 times that of compressive strain, closely aligning with the Poisson's ratio of the aluminum alloy sample. In comparison to other classic NIR ML materials, Sr_3_Sn_1.98_Sb_0.02_O_6.99_ exhibits higher ML intensity and lower response threshold (Figure 5d), highlighting its exceptional capability for microstrain detection. According to the COMSOL simulation results for the aluminum alloy sample, a longitudinal tensile strain of 160 µst induces a transverse compressive strain of 52.8 µst. This value exceeds the ML threshold for compressive strain, thus facilitating the detection of a measurable ML signal, which likely accounts for the observed sensitivity threshold of 160 µst under tensile strain. As the strain gradually increases, the ML intensity under compressive strain significantly surpasses that under tensile strain at the same absolute strain values. At an absolute strain of 200 µst, the ML intensity corresponding to compressive strain is 3.6 times larger than the value corresponding to tensile strain, while this ratio decreases to 2.7 times at an absolute strain of 1500 µst. Furthermore, Figure S19 (Supporting Information) demonstrates favorable microstrain biosensing capability of the Sr_3_Sn_1.98_Sb_0.02_O_6.99_ film. Notably, a 120 µst compressive strain is sufficient to yield to a distinct ML signal through the porcine skin, whereas the detection threshold for tensile strain rises to 460 µst. The ML response threshold of Sr_3_Sn_1.98_Sb_0.02_O_6.99_ through 4 mm of porcine tissue outperforms the previously reported lowest ML response threshold (LiTaO_3_:Tb^3+^, 500 µst). Under 20 charging‐compression strain cycles at 1000 µst, the ML intensity of the sample remains highly stable, indicating good stability and recoverability (Figure 5e).

**Figure 6:**
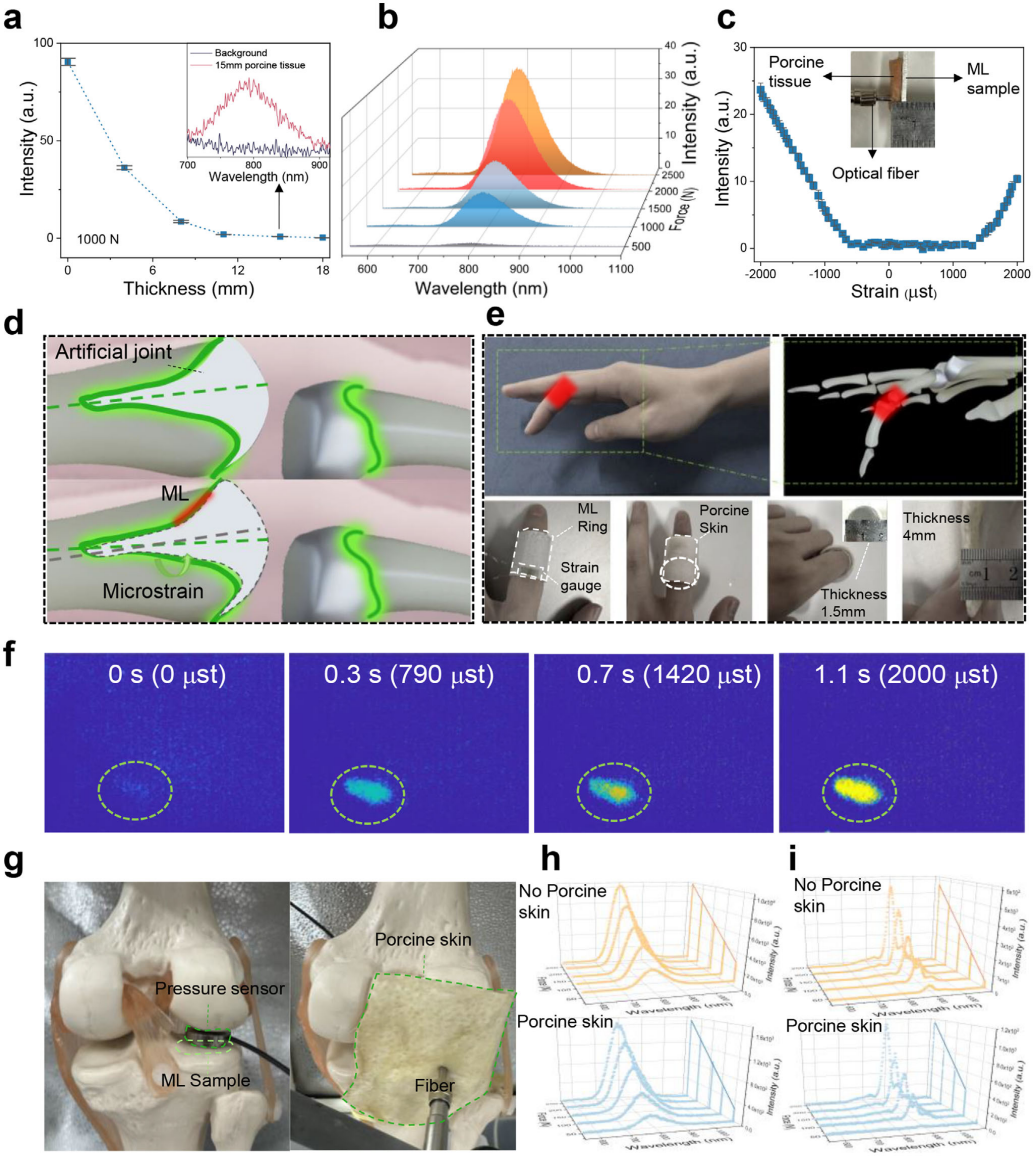
a) ML intensity of the Sr_3_Sn_1.98_Sb_0.02_O_6.99_ sample detected through porcine tissue. Inset showing a magnified view of the ML spectrum. b) ML sensing map detected through 4 mm of porcine tissue. c) Microstrain curves of ML intensity detected through 4 mm of porcine tissue. Charged with 650 nm light through tissue for 1 min. The inset shows the testing setup. d) Schematic of microstrain during the bending of an artificial joint. e) Schematic diagram of the biological sensing system for finger joints. f) Sequential images captured through 4 mm of biological tissue using an NIR camera. g) The bioimaging force test system with and without a 4 mm thick porcine skin. In situ ML spectra of h) Sr_3_Sn_1.98_Sb_0.02_O_6.99_ and i) Sr_3_Sn_2_O_7_:Nd^3+^ before and after passing through porcine skin on artificial joint surfaces.

In 2024, Li et al. demonstrated in situ stress monitoring of a knee joint prosthesis using a NIR ML material of Sr_3_Sn_2_O_7_: Yb^3+^, Nd^3+[^ ^12a^ ^]^ However, the use of a 365 nm LED as the excitation source suffers from poor tissue penetration, and the study did not evaluate the material's sensitivity to microstrain, which limits its applicability in practical, non‐destructive biomedical diagnostics. To further explore the potential of Sr_3_Sn_1.98_Sb_0.02_O_6.99_ for in vivo stress sensing, we conducted a series of non‐destructive in vitro stress sensing and visualization experiments. As shown in **Figure** **6**a, the sample was pre‐irradiated by using red light (650 nm) through layers of porcine tissue of varying thickness, followed by detection of ML through the same tissue thickness. Figure S20a (Supporting Information), illustrates the experimental setup for charging the sample with 650 nm light through the tissue. These results indicate that as the tissue thickness increases, the detected ML signal decreases significantly. Clear ML signals were observed even through 15 mm of tissue (inset of Figure 6a). However, at a thickness of 18 mm, the threshold for in vitro detection was reached, and no distinct ML signal could be detected. Subsequently, we performed charging and detection through 4 mm of porcine tissue while varying the applied compressive stress to evaluate the feasibility of in vitro stress sensing. The ML intensity increased with the applied stress, demonstrating excellent linearity between the stress and ML intensity in the range of 500–2500 N (Figure 6b; Figure S20b, Supporting Information). Further, microstrain sensing experiments were conducted using 650 nm red light for in vitro excitation. A thin ML film affixed to an aluminum alloy tensile specimen was pre‐irradiated and detected through 4 mm of porcine tissue during the tensile process (Figure 6c). The results show that a clear ML signal was detected at a compressive strain of 750 µst, while tensile strains of at least 1580 µst were required to produce detectable ML signals. This represents the first instance of detecting a distinct ML signal at a strain as low as 750 µst using non‐invasive in vitro charging of ML materials. These findings provide a feasible pathway for the application of ML materials in non‐destructive stress sensing within biological systems.

We conducted a case‐study involving stress detection at finger joint, as illustrated in Figure 6d,e. A ML ring was created by mixing Sr_3_Sn_1.98_Sb_0.02_O_7_ powder with epoxy resin in a 1:2 mass ratio into a ring with a thickness of 1.5 mm, comparable to the 1–5 mm thickness of prosthesis adhesive used in joint replacement surgeries. This sensor was designed to monitor forces at the joint. The ring was fitted onto the finger and entirely covered with a 4 mm thick layer of porcine skin. During finger joint movement, clear ML images were captured through the porcine skin (Video S2, Supporting Information), with the processed luminescence intensity map shown in Figure 6f. As strain on the joint increased, the luminescent region became increasingly pronounced, and a strong proportional relationship was observed between the extracted light intensity values and the applied strain. These findings indicate the material's promising potential for non‐invasive stress imaging in vitro. To further validate the joint stress sensing performance of the materials, stress during joint movement was measured using a pressure sensor, and the corresponding ML spectra were collected with an optical fiber spectrometer. The principle of force assessment is illustrated in Figure 6g. A 2‐mm‐thick ML composite layer was affixed to the existing bone structure using adhesive. In situ ML spectra were recorded for two materials: Figure 6h, Sr_3_Sn_1.98_Sb_0.02_O_6.99_ and Figure 6i, Sr_3_Sn_2_O_7_: Nd^3+^. Both exhibited excellent linearity between intensity and stress. After passing through 4 mm of porcine skin, no ML signal was detected from Sr_3_Sn_2_O_7_:Nd^3+^ under 50 N stress, while Sr_3_Sn_1.98_Sb_0.02_O_6.99_ still emitted a clear signal under the same stress. These results demonstrate the superior stress detection threshold of Sr_3_Sn_1.98_Sb_0.02_O_6.99_ and its potential for non‐invasive in vivo stress sensing.

## 3 Conclusion

In summary, we have developed a microstrain sensitive self‐activated broadband NIR ML material of Sr_3_Sn_1.98_Sb_0.02_O_6.99_. We confirmed that ML can be driven by direct defect emission, paving new avenues for understanding ML mechanisms and advancing the development of high‐performance luminescent materials. Benefiting from the rich synergistic interactions among its defects and a minimized energy transfer process, Sr_3_Sn_1.98_Sb_0.02_O_6.99_ exhibits the highest ML intensity and an exceptionally low compressive strain response sensitivity of 50 µst (corresponding to a mere 0.05‰ deformation) along with remarkable stability and repeatability. Charging and detection can be achieved using 650 nm light in vitro. After charging through 15 mm of porcine tissue, ML signals can still be detected through the same tissue thickness. In addition, charging and detecting through 4 mm of porcine tissue allowed the ML signal to be detected at a compressive strain as low as 750 µst. Notably, in biological sensing experiments, this material successfully enabled in situ stress imaging, generating obvious ML signals through 4 mm of porcine skin under the simple bending motion of a finger joint. Furthermore, the sensor exhibits long‐term stability, resistance to compression cycling, saline immersion, and thermal shock. However, in vivo aging and fatigue testing remain challenging. Future work will aim to further demonstrate the material's durability and potential for biological applications in living systems. This study provides innovative insights into the origins of ML and the development of novel defect‐based luminescent materials, marking a significant and pioneering advancement in the field of joint replacement surgery, with substantial potential to improve patient outcomes.

## Conflict of Interest

The authors declare no conflict of interest.

## Author Contributions

W.L. and P.X. contributed equally to this work. W.H.L., J.R. and P.X.X. concepted the experiment. W.H.L., P.X.X., X.X.Z., L.Y.N., L.G.C., Q.Y.W., B.V., P.D. and J.R. performed data curation. W.H.L., B.V., P.D., J.R. and P.X.X. wrote the original draft. P.X. X., J.Z. Z. and J. R. performed visualization. P.X.X. and J.R. performed supervision. All authors contributed to data analysis, discussions and manuscript preparation.

## Supporting information



Supporting Information

Supplemental Video 1

Supplemental Video 2

Supplemental Video 3

Supplemental Video 4

## Data Availability

The data that support the findings of this study are available from the corresponding author upon reasonable request.
