# Supplementary material for: Micro‐Strain Responsive Near‐Infrared Mechanoluminescence for Potential Nondestructive Artificial Joint Stress Imaging
Source: Adv Mater. 2025 Sep 8;38(1):e05360. doi: 10.1002/adma.202505360 (PMC12759234; doi:10.1002/adma.202505360)
Supplement: Supplementary file 1 — Supporting Information [file ADMA-38-e05360-s003.docx]

**Supplementary information**

**Micro-strain responsive near-infrared mechanoluminescence for potential nondestructive artificial joint stress imaging**

Wenhao Li ^1, #^, Puxian Xiong ^2, #,^ *, Xiaoxin Zheng ^1^, Luyue Niu ^3^_,_ Lugui Cui ^1^, Qingyu Wang ^4^, Bruno Viana ^5^, Pieter Dorenbos ^6^, Jianzhong Zhang ^1,^ * and Jing Ren ^1,^ *

^1^ Key Laboratory of In-Fiber Integrated Optics of Ministry of Education, College of Physics and Optoelectronic Engineering, Harbin Engineering University, Harbin 150001, China.

^2^ Department of Electrical and Electronic Engineering, The University of Hong Kong, Hong Kong, 999077, China.

^3^ Macau Institute of Materials Science and Engineering (MIMSE), Macau University of Science and Technology, Taipa 999078, Macao, China.

^4^ College of Nuclear Science and Technology, Harbin Engineering University, Harbin 150001, China.

^5^ PSL Research University Chimie Paris Tech IRCP CNRS Paris 75005, France.

^6^ Faculty of Applied Sciences, Department of Radiation Science and Technology, Delft University of Technology, Mekelweg 15, 2629JB Delft, The Netherlands.

^#^ Equally contributed to this work.

*Corresponding author.

E-mail: pxxiong@hku.hk (P.X. Xiong)

zhangjianzhong@hrbeu.edu.cn (J. Zhang)

ren.jing@hrbeu.edu.cn (J. Ren)

## Computational details

Supplementary **Table 1** Comparison of lattice constants of Sr_3_Sn_2_O_7_ between our calculations and experimental values reported in the literature

|  | Sr_3_Sn_2_O_7_ | Sr_3_Sn_2_O_7_ (in exp.^[1]^) |
| --- | --- | --- |
| a | 5.82 Å | 5.73 Å |
| b | 5.81 Å | 5.71 Å |
| c | 20.89Å | 20.66Å |
| α/β/γ | 90^o^ | 90^o^ |

The formation energy of doping ion *X* with the charge state *q* dependent on the Fermi level position were calculated according to^[2]^

$$E^{f}[X^{q}]=E_{tot}[X^{q}]-E_{tot}[bulk]-\sum_{i} n_{i}\mu_{i}+q(E_{F}+E_{V}+Ecorr)$$

Where *E_tot_*[*X^q^*] is the total energy of the supercell with defect states charged with *q*. *E_tot_*[*bulk*] is the initial supercell without defects. *n_i_* is the number of atoms of species *i* that have been added or removed, and *μ_i_* refers to the corresponding chemical potentials. *E_F_* represents the Fermi level with reference to the bulk VBM *E_v_*. *E_corr_* is a comprehensive correction term^[3]^, aligning the reference potential of the defects-containing supercell with that of the perfect bulk.

For Sr_3_Sn_2_O_7_, *μ_Sr_*≤-1.60 eV, *μ_Sn_*≤-4.83 eV and *μ_O_*≤-7.84 eV are satisfied to avoid the solid/gas precipitation.

In order to produce Sr_3_Sn_2_O_7_, the *μ_i_* of all three of them should satisfy:

$${3\mu}_{Sr}+{2\mu}_{Sn}+7\mu_{O}=\mu_{{Sr}_{3}{Sn}_{2}O_{7}}$$

In fact, SrO, SnO_2_ may be generated in the formation process of Sr_3_Sn_2_O_7._ Thus, the following conditions should also be satisfied,

$$\mu_{Sr}+\mu_{O}\leq\mu_{SrO}$$

$$\mu_{Sn}+2\mu_{O}\leq\mu_{SnO_{2}}$$

## Experiment Section

***Synthesis of Sr_3_Sn_2-x_Sb_x_O_7-x/2_ Phosphors****:* Phosphor samples were synthesized according to the chemical formulation of Sr_3_Sn_2-x_Sb_x_O_7-x/2_ (x=0%, 1%, 2%, 3%, 5% and 10%). Additionally, 2 mol.% of H_3_BO_3_ and 5 mol.% of Li_2_CO_3_ were incorporated. The synthesis was conducted through solid-state reaction. High-purity compounds including SrCO_3_(99.99%), SnO_2_(99.99%), Sb_2_O_3_(99.99%), Li_2_CO_3_(99.99%), and H_3_BO_3_(99.99%) were accurately weighed and subsequently ground in an agate mortar for 10 min. This prepared mixture was then transferred to a corundum crucible for further processing. The sintering process was carried out at a temperature of 1500 °C for a duration of 5 hours, under a highly pure N_2_ atmosphere. This controlled environment ensured the optimal formation and integrity of the samples. After cooling the samples to room temperature, they were thoroughly ground to ensure uniformity for subsequent testing.

***Synthesis of Sr_3_Sn_2_O_7_:Nd^3+^ Phosphors****:* Phosphor samples were synthesized according to the chemical formulation of Sr_2.99_Sn_2_O_7_:0.01Nd^3+^. The synthesis was conducted through solid-state reaction. High-purity compounds including SrCO_3_(Aladdin, 99.99%), SnO_2_(Aladdin, 99.99%), H_3_BO_3_(Aladdin, 99.99%), and Nd_2_O_3_(Aladdin, 99.99%) were accurately weighed and subsequently ground in an agate mortar. This prepared mixture was then transferred to a corundum crucible for further processing. The sintering process was carried out at a temperature of 1500 °C for a duration of 5 hours, under a highly pure N_2_ atmosphere. After cooling the samples to room temperature, they were thoroughly ground to ensure uniformity for subsequent testing.

***Synthesis of Sr_3_Sn_2_O_7_:Sm^3+^ Phosphors****:* Phosphor samples were synthesized according to the chemical formulation of Sr_2.98_Sn_2_O_7_:0.02Sm^3+^. The synthesis was conducted through solid-state reaction. High-purity compounds including SrCO_3_(Aladdin, 99.99%), SnO_2_(Aladdin, 99.99%), H_3_BO_3_(Aladdin, 99.99%), and Sm_2_O_3_(Aladdin, 99.99%) were accurately weighed and subsequently ground in an agate mortar. This prepared mixture was then transferred to a corundum crucible for further processing. The sintering process was carried out at a temperature of 1500 °C for a duration of 5 hours, under a highly pure N_2_ atmosphere. After cooling the samples to room temperature, they were thoroughly ground to ensure uniformity for subsequent testing.

***Synthesis of CaZnOS:Nd^3+^, Mn^2+^ Phosphors****:* Phosphor samples were synthesized according to the chemical formulation of Ca_0.995_Zn_0.995_OS:0.005Nd^3+^, 0.005Mn^2+^. The synthesis was conducted through solid-state reaction. Additionally, 6 mol.% of Li_2_CO_3_ were incorporated. High-purity compounds including CaCO_3_ (Aladdin, 99.99%), ZnS (Bide, 99.99%), Nd_2_O_3_ (Aladdin, 99.99%), MnO (Aladdin, 99.99%) and Li_2_CO_3_ (Aladdin, 99.99%) were accurately weighed and subsequently ground in an agate mortar. This prepared mixture was then transferred to a corundum crucible for further processing. The sintering process was carried out at a temperature of 1100 °C for 3 h under an argon atmosphere. After cooling the samples to room temperature, they were thoroughly ground to ensure uniformity for subsequent testing.

***Synthesis of LiNbO_3_:Nd^3+^ Phosphors****:* Phosphor samples were synthesized according to the chemical formulation of Li_0.98_NbO_3_:0.02Nd^3+^. The synthesis was conducted through solid-state reaction. High-purity compounds including Li_2_CO_3_ (Aladdin, 99.99%), Nb_2_O_5_ (Aladdin, 99.99%) and Nd_2_O_3_ (Aladdin, 99.99%) were accurately weighed and subsequently ground in an agate mortar. This prepared mixture was then transferred to a corundum crucible for further processing. The sintering process was carried out at a temperature of 1050 °C for 8 h under an air atmosphere. After cooling the samples to room temperature, they were thoroughly ground to ensure uniformity for subsequent testing.

***Synthesis of LiTaO_3_:Tb^3+^ Phosphors****:* Phosphor samples were synthesized according to the chemical formulation of Li_0.994_NbO_3_:0.006Tb^3+^. The synthesis was conducted through solid-state reaction. High-purity compounds including Li_2_CO_3_ (Aladdin, 99.99%), H_3_BO_3_(Aladdin, 99.99%), Ta_2_O_5_ (Aladdin, 99.99%) and Tb_4_O_7_ (Aladdin, 99.99%) were accurately weighed and subsequently ground in an agate mortar. This prepared mixture was then transferred to a corundum crucible for further processing. The sintering process was carried out at a temperature of 1050 °C for 8 h under an air atmosphere. After cooling the samples to room temperature, they were thoroughly ground to ensure uniformity for subsequent testing.

***Preparation of ML Pellet:*** As to ML measurements, the powders were sieved through a 200-mesh screen and mixed homogeneously with a commercial optical epoxy resin (Shenzhen Juhengchuang Electronic Materials Co., Ltd., Model 7737) with a mass ratio of 1:4. After mixing, the colloids were transferred to a columnar silicone mold (Ф = 25 mm, h = 10 mm) and solidified in a drying oven at 70 °C for 2 hours. Similarly, the synthetic Sr_3_Sn_2_O_7_: Nd^3+^, CaZnOS: Nd^3+^ and Li_2_CO_3_: Nd^3+^ phosphors were also made into elastic pellets in the same way

***Fabrication of ML Film and Aluminum Alloy Sheet****:* In order to clarify the ML responses of the Sr_3_Sn_2-x_Sb_x_O_7-x/2_ phosphor at low strain levels, silkscreen printing technology was employed to produce ML films and aluminum alloy sheets. First, according to a mass ratio of 8.8:1, 8.80 g of Sr_3_Sn_1.98_Sb_0.02_O_7_ phosphor and 1.00 g of epoxy resin were weighed in a beaker. Subsequently, 0.5 g of curing agent was added to promote curing. After 5 min of uniform stirring by glass rod, the obtained white thick slurry could be printed on aluminum foil (thick: 20 μm) to fabricate an ML film (1 × 1 cm, thick: 120 μm) by screen printing technology. Finally, the as-prepared film was transferred into an oven at 70 °C for a duration of 2 h. Similarly, the synthetic Sr_3_Sn_2_O_7_: Nd^3+^, CaZnOS: Nd^3+^ and LiNbO_3_: Nd^3+^ phosphors were also fabricated into thin films using the same method. At the end of curing and cooling, the ML film was pasted on the central area of the aluminum alloy sheet using strain gauge glue (CC-33A, 30501, Japan). Simultaneously, it is necessary to apply pressure to the surface using a steel ruler for at least 5 min to ensure tight bonding between the film and the aluminum alloy sheet. In the same way, the strain gauge must also be pasted tightly on the other face of the aluminum alloy sheet to detect the strain transmitted from the aluminum alloy sheet to the ML film in real time.

**Characterizations**

X-ray diffraction patterns (XRD) were measured by an X-ray diffractometer (D/MAX 2550VB/PC, Rigaku Corporation, Japan) with the Cu-Kα irradiation. Microscopic images were recorded by SEM (Apero S, Thermo Scientific) equipped with an energy disperse spectrometer (EDS, Apero S, Thermo Scientific), and by TEM (FEI Talos F200x, American FEI Company). XPS (Thermo SCIENTIFIC Nexsa) was equipped with a focused monochromatic Al Kα X-ray beam to examine the trap defects. A home-made ML measurement system was applied which mainly contains three parts: an excitation light source, a universal test machine (SUST CMT 1104, China) and two highly sensitive CCD spectrometer (Horiba Lumetta, Canada and Ocean Optics fiber spectrometer NIRQuest1.7). The excitation sources included a 365 nm ultraviolet (UV) LED with an output power of 12 W, a 650 nm LED with an output power of 12 W, and an X-ray tube (MOXTEK TUB-MAN-1006). Samples were irradiated at a distance of 20 mm from the light source, and the ML signals were collected by a large-core (d = 1000 μm, N.A. = 0.22) silica fiber in a dark room. TL glow curves from room temperature to 500 °C was recorded 1 min later after the UV irradiation by a TL glow curves dosimeter (LTTL-3DS, GUANGZHOU RONGFAN TECHNOLOGY CO., Ltd., China) with a heating rate of 2 °C/s and then left for 2 min. The PL/PLE spectra, luminescence decay time were recorded using the Edinburgh FLS1000 fluorescence spectrometer equipped with a 450 W Xe lamp. Electron paramagnetic resonance (EPR) measurements were carried out using a JEOL JESX320 model spectrometer (Tokyo, JPN) (Test parameters: microwave frequency (9.83 GHz), 2 mW microwave power). The diffuse reflection spectra (DRS) were measured by a UV-Vis-NIR Spectrophotometer 5000. NIR photos were taken by a NIR camera (SPLG-Mars640, 900-1700 nm SPLG Shenzhen Photonics Co., Ltd., China). Unless specially stated, all the data were collected at room temperature.

## Calculation Section

The structure determination of Sr_3_Sn_2_O_7_ was performed using the projector augmented wave (PAW) method^[4]^ within the framework of the Vienna Ab initio Simulation Package (VASP) code^[5]^, which operates based on density functional theory (DFT). For the plane wave basis functions, an energy cutoff of 500 eV was applied. The Perdew-Burke-Ernzerhof (PBE) functional^[6]^ was employed for structure relaxations, while the Heyd-Scuseria-Ernzerhof (HSE) functional^[7]^, operating under the generalized gradient approximation (GGA), was utilized for electron structure calculations^[8]^. The valence states for Sr (4*s*²4*p*⁶5*s*²), Sn (5*s*²5*p*²), and O (2*s*²2*p*⁴) were considered. Sampling of the Brillouin zone utilized a Monkhorst–Pack scheme with a 2×6×6 K-point mesh^[9]^. Convergence of the structural optimization was achieved when the Hellmann–Feynman forces on all atoms fell below 10^-2^ eV/Å. Both structural optimization and electronic structure calculations employed the self-consistent field (SCF) method, with a convergence threshold of 10^-5^ eV. Sr_3_Sn_2_O_7_ crystals of A2_1_am space group was used for all calculations to ensure the agreement with the experiment. After the structure relaxation, the crystal structure parameters were obtained as Supplementary Table 1, consistent with the results of the literature^[1, 10]^.

Supplementary **Table 2** Chemical potentials of each element in Sr_3_Sn_2_O_7_ under Sr-rich and Sr-deficient conditions

|  | Sr-rich | Sr-deficient |
| --- | --- | --- |
| *μ_Sr_* | -7.48 eV | -3.65 eV |
| *μ_Sn_* | -10.63 eV | -10.11 eV |
| *μ_O_* | -7.84 eV | -9.63 eV |

Supplementary **Table 3** Linear fitting of ML intensity as a function of applied load for Sr_3_Sn_1.98_Sb_0.02_O_6.99_, Sr_3_Sn_2_O_7_: Nd^3+^, CaZnOS: Nd^3+^, and LiNbO_3_: Nd^3+^ samples

| **Samples** | **a** | **b** | **R^2^** |
| --- | --- | --- | --- |
| Sr_3_Sn_1.98_Sb_0.02_O_6.99_ | 3.91 | 0.0515 | 0.997 |
| Sr_3_Sn_2_O_7_: Nd^3+^ | 1.52 | 0.019 | 0.996 |
| CaZnOS: Nd^3+^ | 0.21 | 0.0023 | 0.997 |
| LiNbO3: Nd^3+^ | 0.16 | 0.0018 | 0.982 |

**Fig. S1.** ML spectra of (a) Sr_3_Sn_1.98_Sb_0.02_O_6.99_ and (b) Sr_3_Sn_1.98_X_0.02_O_6.99_ (X=In, Si, Ge, P, As and Bi) under a compressive force of 1000 N at room temperature. Samples were irradiated with 365 nm UV LED for 1 min and measured after 1 min in the dark. (c) XRD of Sr_3_Sn_1.98_X_0.02_O_6.99_ (X=In, Si, Ge, P, As and Bi).

A series of Sb^3+^ doped Sr_3_Sn_2_O_7_ phosphors, denoted as Sr_3_Sn_2-x_Sb_x_O_7-x/2_ were synthesized utilizing solid-state reaction with optimized amounts of fluxing agents (H_3_BO_3_ and Li_2_CO_3_) in a N_2_ atmosphere. The substitution of Sn with Sb was motivated by the observation of a consistent "defect emission peak" in Nd^3+^ and Sm^3+^ doped Sr_3_Sn_2_O_7_ phosphors. This prompted an exploration of Sn substitution with elements from the same period and group, such as Sb, to introduce defects and modulate the bandgap with the goal of activating this promising defect-related peak. Following the introduction of these non-rare-earth ions, defect emission was successfully observed with Sb-doping, which

became the focus of subsequent studies (Fig. S1, Supporting Information).

**Fig. S2.** Rietveld refinement of Sr_3_Sn_2-x_Sb_x_O_7-x/2_ (*x* = 0, 1, 2, 3, 5, 10 mol.%). The black crosses and red solid lines represent the experimental and calculated patterns, respectively. Vertical bars indicate the Bragg peak positions, and the blue line at the bottom shows the residuals.

**Fig. S3.** (a) PLE of Sr_3_Sn_2-x_Sb_x_O_7-x/2_ (*x* = 1, 2, 3, 5, 10 mol.%). (b) PL spectra of Sr_3_Sn_2-x_Sb_x_O_7-x/2_ (*x* = 0, 1, 2, 3, 5, 10 mol.%). (c) Fluorescence lifetime decay curves of Sr_3_Sn_2-x_Sb_x_O_7-x/2_ (*x* = 1, 2, 3, 5, 10 mol.%). (d) Fluorescence lifetime values of Sr_3_Sn_2-x_Sb_x_O_7-x/2_ (*x* = 1, 2, 3, 5, 10 mol.%).

Notably, the position of the excitation peak in the PLE spectra with different Sb^3+^ content does not change significantly (Fig. S3a, Supporting Information). Under 254 nm optical excitation, Sr_3_Sn_2-x_Sb_x_O_7-x/2_ (x = 0.0-10.0 %) exhibits broadband NIR emission in the range of 700-1000 nm (FWHM > 100 nm) (Fig. S3b, Supporting Information). The strongest PL intensity is observed when x = 1%, and as x increases further, the PL intensity gradually decreases. Room-temperature luminescence decay curves (λ_ex_ = 254 nm, λ_em_ = 800 nm) were measured for various samples (Fig. S3c and S3d, Supporting Information). The measured lifetimes is in the order of several hundred microseconds, significantly longer than the typical lifetimes of Sb^3+^-based luminescent materials, which are usually in the tens of microseconds^[11]^. As Sb^3+^ concentration increases, PL lifetime decreases from 807 μs to 429 μs. This decrease is primarily attributed to concentration quenching caused by the increased defect density.

**Fig. S4.** (a) ML spectra of Sr_3_Sn_2-x_Sb_x_O_7-x/2_ with different x values under a compressive force of 1000 N at room temperature. ML spectra of Sr_3_Sn_1.98_Sb_0.02_O_7_ doped with varying concentrations of (b) H_3_BO_3_ and (c) Li_2_CO_3_ under a compressive force of 1000 N at room temperature. Samples were pre-irradiated with a 365 nm UV LED for 1 min and measured after a 1 min dark interval. The ML intensity versus (d) Sb^3+^, (e) H_3_BO_3_ and (f) Li_2_CO_3_ doping concentration.

Fig. S4 (Supporting Information) shows the ML spectra of Sr_3_Sn_2-x_Sb_x_O_7-x/2_ phosphors varying Sb^3+^ concentrations and different concentrations of H_3_BO_3_ and Li_2_CO_3_ under a compressive force of 1000 N. The ML spectra reveal a broad near-infrared emission band spanning in the range 650-1000 nm, with a prominent peak at 796 nm and a full width at half maximum (FWHM) of 111 nm. As Sb is introduced into the samples, ML signals emerged from nothing and rapidly increased in intensity, reaching its maximum at a doping concentration of 2 mol%. Raman spectroscopy reveals the structural evolution before and after doping (Fig. S5, Supporting Information). For Sr_3_Sn_2-x_Sb_x_O_7-x/2_ samples, the strongest peak at 597.6 cm^-1^ corresponds to the stretching mode of SnO_6_ octahedra^[12]^. The low-frequency modes in the range of 120-190 cm^-1^ are attributed to rotations around the c-axis, tilts around the a-axis of SnO_6_ octahedra, and vibrations of Sr and Sn atoms. As the Sb doping concentration exceeds 3 mol%, the intensity of these modes gradually decreases, indicating a reduction in Sr ion vibrations along the b-axis and Sn ion vibrations along the c-axis, which are associated with the phonons in this range^[13]^. When the Sb doping concentration reaches 3 mol%, new peaks emerge in the ranges of 75-95 cm^-1^, 195-235 cm^-1^, and 557-588 cm^-1^. These new peaks suggest that increased doping induces lattice distortions, causing abrupt changes in vibrational frequencies. This implies that the material enters an unstable phase transition, likely involving a coexistence of orthorhombic and tetragonal phases. The observed decrease in ML intensity at this point is attributed to the disruption of the orthorhombic crystal field environment^[14]^.

**Fig. S5.** (a) Raman spectrum and (b) normalized Raman spectra of Sr_3_Sn_2-x_Sb_x_O_7-x/2_ varying x values.

**Fig. S6.** XRD of Sr_3_Sn_2_O_7_, Sr_3_Sn_1.98_Sb_0.02_O_6.99_, Sr_3_Sn_2_O_7_: Nd^3+^ and Sr_3_Sn_2_O_7_: Sm^3+^. The standard card of Sr_3_Sn_2_O_7_ (PDF #25-0914) is provided at the bottom.

**Fig. S7.** ML spectra of Sr_3_Sn_1.98_Sb_0.02_O_6.99_, Sr_3_Sn_2_O_7_:Nd^3+^ and Sr_3_Sn_2_O_7_:Sm^3+^ under a compressive force of 1000 N at room temperature. Samples were pre-irradiated with a 365 nm UV LED for 1 min and measured after a 1 min dark interval.

**Fig. S8.** Schematic energy transfer mechanisms for Sr_3_Sn_2_O_7_:Nd^3+^ and Sr_3_Sn_2_O_7_:Sm^3+^.

**Fig. S9.** (a) Set up used for the ML measurements. (b) Temperature-controlled ML measurement setup.


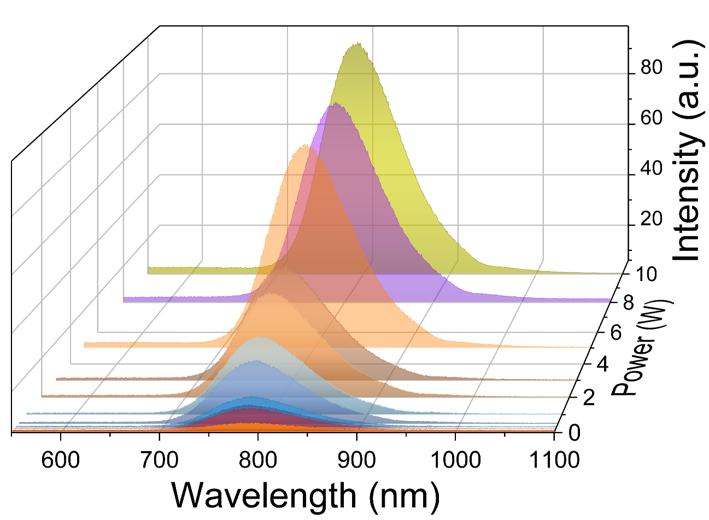


**Fig. S10.** 3D ML spectra of Sr_3_Sn_1.98_Sb_0.02_O_6.99_ under 1000 N stress induced by different X-rays power levels (0.045, 0.06, 0.08, 0.1, 0.2, 0.3, 0.5, 1, 2, 3, 5, 8 and 10W). Samples were pre-irradiated with X-rays for 1 min and measured after a 1 min dark interval.

**Fig. S11.** Spectra recorded of (a) sunlight and (b) moonlight as used for the proposed experiments.

**Fig. S12.** (a) Variation in ML peak intensity recorded over the first 10 cycles of continuous mechanical loading. (b) ML peak intensity changes over 90 subsequent loading cycles. (b) ML Spectrum at the 100th Continuous Compression. Samples were subjected to a compressive force of 1000 N at room temperature, irradiated with a 365 nm UV LED for 1 min, and measured after a 1 min dark interval.

**Fig. S13** (a) TL glow curves of Sr_3_Sn_2-x_Sb_x_O_7-x/2_ (*x* = 0, 1, 2, 3, 5, 10 mol.%). (b) The diffuse reflectance spectrum of the Sr_3_Sn_2-x_Sb_x_O_7-x/2_ (*x* = 0, 1, 2, 3, 5, 10 mol.%) samples. (c) Variations of PAL (long τ_1_ and short τ_2_ obtained by fittings) as a function of the Sb^3+^ concentration. (d) TL peak positions of Sr_3_Sn_2-x_Sb_x_O_7-x/2_ samples with varying Sb^3+^ concentrations. (e) Estimated optical bandgap energies of Sr_3_Sn_2-x_Sb_x_O_7-x/2_ as a function of Sb^3+^ doping concentration. (f) PAL spectra showing variations in lifetime components with increasing Sb^3+^ content in Sr_3_Sn_2-x_Sb_x_O_7-x/2_. (g) EPR curves of Sr_3_Sn_2-x_Sb_x_O_7-x/2_ regarding g-factor. (h) High-resolution XPS spectra of O 1s and Sb 3d with diffident Sb ratio. (i) The percentage of different oxygen components in the high-resolution XPS spectrum O 1s at different Sb concentration.

TL glow curves were investigated as a function of Sb doping concentrations (Fig. S13a and S13d, Supporting Information). The TL glow curve of the undoped sample shows no detectable TL glow peaks, indicating an insufficient trap concentration to get a TL signal. However, with the incorporation of Sb, prominent TL glow peaks appear around peak 1, peak 2, and peak 3, signifying that Sb doping introduces a wealth of defect energy levels. Peak 2 appears to be a typical TL glow peak, while peaks 1 and 3 exhibit significant broadening. Peak 2 likely involves thermal excitation across an energy barrier, corresponding to the trap depth. In contrast, the broad nature of peaks 1 and 3 may be attributed to a temperature-assisted tunneling recombination mechanism, where trapped holes and electrons recombine as neighboring pairs.^[15]^ The TL glow peak near 360 K corresponds to shallow-level defects, primarily contributing to afterglow emission, while the 397 K peak is associated with intermediate-energy level defects, predominantly responsible for ML. The TL glow peak near 537 K corresponds to deep-level defects, where stored electrons can transition to shallower energy levels under the influence of stress or temperature to contribute to ML. As Sb concentration increases, the intensity of these TL glow peaks grows significantly, reaching a maximum at a doping concentration of 2 mol%, which corresponds to the highest defect concentration. Beyond this point, the TL intensity decreased, a trend consistent with the variation in ML intensity, indicating a close correlation between ML and defect levels. Interestingly, after trivalent Sb doping, the positions of the TL glow peaks show a clear correlation with Sb concentration, with all three peaks shifting towards lower temperatures as the doping level increases. This phenomenon can be well explained by the experimental bandgap values derived from the diffuse reflectance spectra in Fig. S13b and S13e (Supporting Information). As shown in the inset, the bandgap decreases with increasing Sb concentration. The bandgap value gradually reduces from 4.3 eV to approximately 4.1 eV, corresponding to a decrease of 0.2 eV. This reduction aligns with a shift of 30–40 K in the TL peak positions in Fig. 4a, which corresponds to a trap depth shift of approximately 0.1 eV. Considering that a reduced bandgap is often accompanied by an upward shift of the VB and a downward shift of the CB, the relative positions of defect levels within the bandgap also change accordingly. This results in a correlated trend between the bandgap reduction and the TL peak shifts. The reduced bandgap lowers the energy barrier for electrons delocalizing to the CB and holes ionizing to the VB from defect levels, making it easier for them to be excited into the CB/VB and subsequently emit light. Consequently, the TL glow peaks shift toward lower temperatures.

To further elucidate the effect of Sb^3+^ doping on the concentration and distribution of defects in Sr_3_Sn_2_O_7_ compounds, positron annihilation lifetime (PAL) spectroscopy was performed. As shown in Fig. S13c and S13f (Supporting Information), samples with varying Sb concentrations exhibit comparable PAL spectra. The spectra decompose into two main lifetime components: a short lifetime (*τ*_1_), associated with positron interactions with smaller vacancies (e.g., single vacancies) or shallower positron traps such as oxygen vacancies^[16]^, and a longer lifetime (*τ*_2_), resulting in a greater number of defects, such as surface oxygen vacancy clusters^[17]^. The relative intensities of these components, denoted as *I*_1_ and *I*_2_, provide information about the defect concentrations. Upon Sb doping, no significant changes are observed in the relative intensities (*I*_1_, *I*_2_). However, *τ*_1_ gradually increases with increasing Sb content, while *τ*_2_ shows a distinct trend: it initially increases significantly with Sb doping, reaching a maximum at 2 mol% Sb, and then gradually decreases as Sb doping levels continue to rise. This indicates that Sb doping not only introduces new defects but also increases the number of existing defects, leading to the formation of more abundant defect clusters. To identify the key defect types influencing ML, we compared Sr_3_Sn_1.98_Sb_0.02_O_6.99_ samples sintered under different atmospheric conditions. The results showed a significant enhancement in ML for samples sintered in inert gas environments. Specifically, samples sintered in nitrogen and argon exhibited ML intensities approximately 2 times and 1.5 times higher, respectively, than those sintered in air (Fig. S14, Supporting Information). This indicates that oxygen-deficient environments can promote the formation of oxygen vacancies. The observed correlations among TL, ML, and *τ*_2_ indicate that ML is closely linked to long-lifetime defects, such as surface oxygen vacancy clusters.

Additionally, to further analyze the chemical environment and defect evolution following Sb doping, electron paramagnetic resonance (EPR) measurements were performed on Sr_3_Sn_2-x_Sb_x_O_7-x/2_ (Fig. S13g, Supporting Information). EPR spectroscopy offers insights into the presence and behavior of impurities, as well as native defects such as vacancies and interstitials. Notably, the characteristic g-factor of approximately 2 corresponds to oxygen vacancy defects formed under high-temperature solid-state reactions^[18]^. X-ray photoelectron spectroscopy (XPS) was employed to exploit the evolution of local chemical environment on Sr_3_Sn_2-x_Sb_x_O_7-x/2_ samples (Fig. S15, Supporting Information). Fig. S13e (Supporting Information) shows that the characteristic peak at 539.8 eV corresponds to the Sb^3+^ 3d_3/2_ level, while the prominent peaks at 531.2 eV and 529.6 eV are attributed to the O 1s and Sb^3+^ 3d_5/2_ levels, respectively^[19]^. The Sb^3+^ 3d_5/2_ peak was successfully separated from the overlapping O 1s peak by deconvolution, based on the characteristic relationship between the Sb 3d_3/2_ and Sb 3d_5/2_ peak positions. By fitting the O 1s peaks near 531.2 eV and 529.6 eV according to their different chemical states, we identified the low-energy peaks in the O 1*s* XPS spectra as originating from lattice oxygen in Sr_3_Sn_2-x_Sb_x_O_7-x/2_, while the high-energy peaks were attributed to interstitial oxygen and oxygen vacancies, respectively. Notably, the proportion of oxygen vacancies in the O 1*s* spectrum increases progressively with rising Sb doping concentration (Fig. S13h and S13i, Supporting Information), reaching a maximum at 2 mol%. In contrast, the amount of interstitial oxygen exhibits an inverse trend, initially decreasing with Sb doping and reaching its lowest point at 2 mol%. The lattice oxygen content generally shows an upward trend with increasing Sb concentration. Interestingly, the variation in oxygen vacancy concentration closely mirrors the trend in ML intensity, suggesting a critical intrinsic relationship between oxygen vacancies and the generation and enhancement of ML. Given the high-temperature, oxygen-deficient conditions during sample preparation, oxygen vacancies readily form and become abundant. As positively charged donor defects, oxygen vacancies are capable of trapping electrons, and under stress, these trapped electrons may be key contributors to ML. Identifying the defect types responsible for ML and understanding their role offers new avenues for the development and design of high-performance ML materials.

**Fig. S14.** ML spectra and intensity of Sr_3_Sn_1.98_Sb_0.02_O_6.99_ samples sintered in nitrogen, argon, and air environments. Samples were subjected to a compressive force of 1000 N at room temperature, irradiated with a 365 nm UV LED for 1 min, and measured after a 1 min dark interval.

**Fig. S15.** Full scanning XPS spectra of Sr_3_Sn_2-x_Sb_x_O_7-x/2_ (*x* = 0, 1, 2, 3, 5, 10 mol.%).

**Fig. S16.** (a) Stability range of the chemical potentials (in eV) of the Sr_3_Sn_2_O_7_ constituents**.** (b) Formation energies (E*_form_*) of various defects in the Sr_3_Sn_2_Sb_0.02_O_6.99_ compound.

**Fig. S17.** Partial density of states for the stable charge states of (a) defects and (b) defect-free Sr_3_Sn_2_O_7_.

**Fig. S18.** The viability of three different types of (a) L929 cells, (b) Huvec cells and (c) MC3T3-E1 cells in different concentrations of Sr_3_Sn_1.98_Sb_0.02_O_6.99_ powder solution.

**Fig. S19.** Microstrain data, measured using a 4 mm pig tissue, recorded after 10 s of charging at 365 nm under room temperature.

**Fig. S20.** (a) Charging process using a 650 nm LED through 4 mm of porcine tissue. (b) Linear fitting of ML intensity versus applied force detected through 4 mm of porcine tissue after 1 min of 650 nm light charging through the same tissue thickness.

**Fig. S21.**
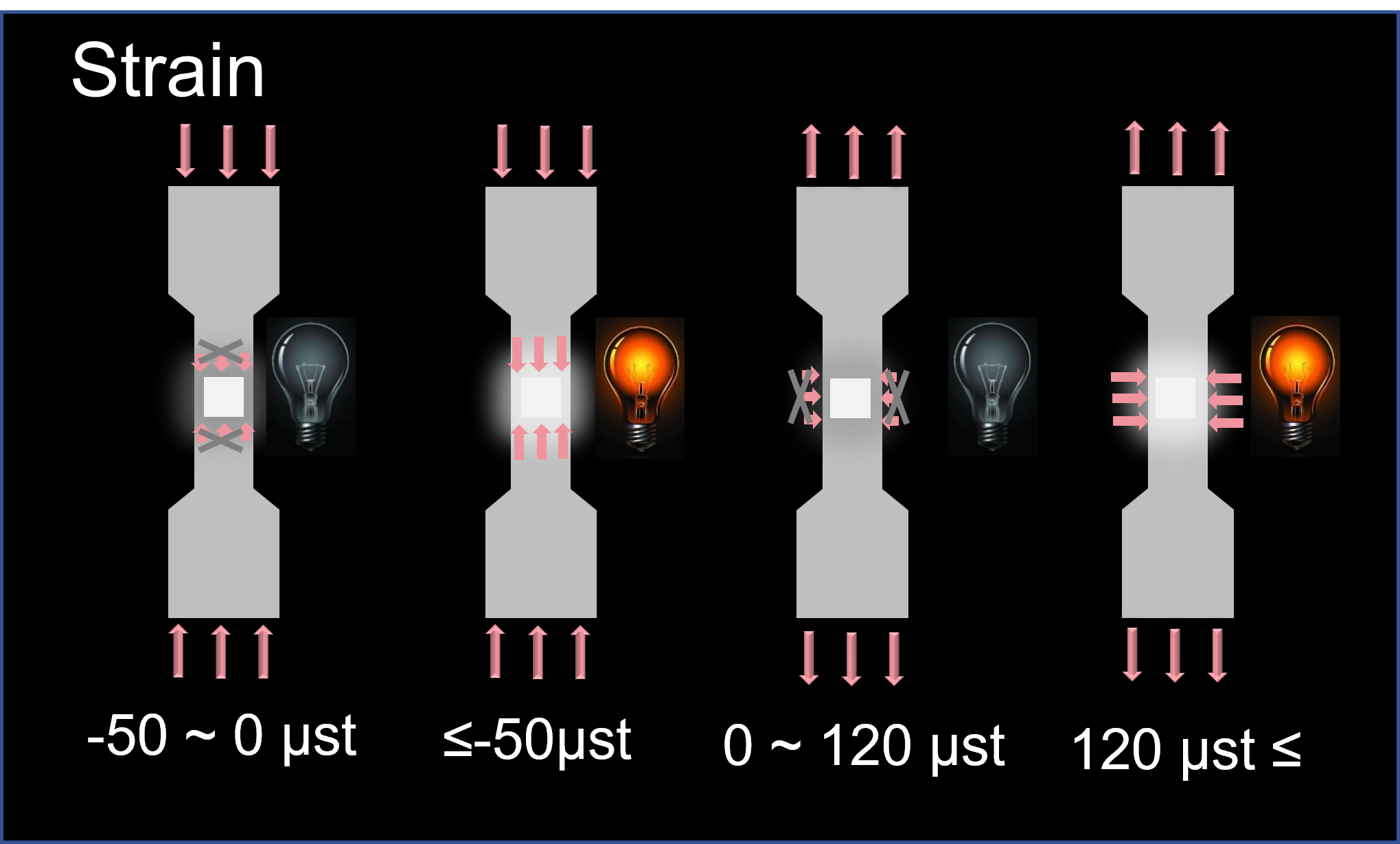
 Correlation between strain direction, magnitude, and ML. No ML signals were detected under compressive strains ranging from -50 μst to 0 μst. However, ML signals became detectable when the compressive strain was less than -50 μst. Similarly, no ML signals were observed under tensile strains between 0 μst and 120 μst, but ML signals could be detected when the tensile strain exceeded 120 μst.

**Fig. S22.** Image of ML from the Sr_3_Sn_1.98_Sb_0.02_O_6.99_ sample during the application of a compressive force (1000 N). (a) before compression (b) during compression (c) at peak compression and (d) after compression. Figures (e-h) correspondingly illustrate the light intensity extraction patterns corresponding to Figures (a-d). Samples were pre-irradiated with a 365 nm UV LED for 1 min and measured after a 1 min dark interval.

**Fig. S23** (a) Comparison of ML spectra from the same Sr_3_Sn_1.98_Sb_0.02_O_6.99_ sample tested in ambient atmosphere. The control spectrum was measured in October 2024, the experimental spectrum was recorded after eight months in June 2025. (b) Water bath stability test: the sample was immersed in normal saline at 310 K (physiological temperature) for 168 h. (c) ML spectra before and after saline immersion. (d) Thermal shock test: the sample was cycled between 253 K (1 h) and 310 K (1 h) for five cycles. (e) ML spectra before and after thermal shock treatment. All samples were irradiated with a 365 nm UV LED for 1 min and measured after 1 min in the dark, followed by testing under a compressive force of 1000 N at room temperature.

The ML stability under repeated compression and temperature variations has been investigated in the paper. We further evaluated the material stability through three tests: long-term storage, normal saline immersion, and thermal shock (Fig. S23, Supporting Information). First, ML spectra of a sample stored under ambient conditions for eight months were remeasured. The results showed good agreement in ML intensity compared with the spectra collected in October 2024 (Fig. S23a, Supporting Information). Second, ML sample was immersed in a 310 K normal saline bath (approximating human body temperature) for 7 days to assess chemical stability (Fig. S23b, Supporting Information). Comparison of ML spectra before and after immersion revealed minimal changes in signal intensity. Third, ML sample was subjected to a 10-hour thermal shock test, alternating between cooling at 253 K for 1 hour and heating at 310 K for 1 hour, repeated five times (Fig. S23c, Supporting Information). The ML intensity before and after the thermal shock remained consistent (Fig. S23d, Supporting Information). Notably, no visible deformation or damage was observed in any of the samples after these tests, and the ML signals remained stable, demonstrating the excellent durability of the material.

## Reference

[1] Y. Wang, F. T. Huang, X. Luo, B. Gao, S. W. Cheong, The First Room‐Temperature Ferroelectric Sn Insulator and Its Polarization Switching Kinetics, *Advanced Materials* **2017**, 29, 1601288.

[2] a)C. G. Van de Walle, J. Neugebauer, First-principles calculations for defects and impurities: Applications to III-nitrides, *Journal of applied physics* **2004**, 95, 3851; b)S. Lany, A. Zunger, Assessment of correction methods for the band-gap problem and for finite-size effects in supercell defect calculations: Case studies for ZnO and GaAs, *Physical Review B* **2008**, 78, 235104.

[3] C. Freysoldt, J. Neugebauer, C. G. Van de Walle, Fully ab initio finite-size corrections for charged-defect supercell calculations, *Physical review letters* **2009**, 102, 016402.

[4] G. Kresse, D. Joubert, From ultrasoft pseudopotentials to the projector augmented-wave method, *Physical review b* **1999**, 59, 1758.

[5] a)G. Kresse, J. Furthmüller, Efficiency of ab-initio total energy calculations for metals and semiconductors using a plane-wave basis set, *Computational materials science* **1996**, 6, 15; b)G. Kresse, J. Furthmüller, Efficient iterative schemes for ab initio total-energy calculations using a plane-wave basis set, *Physical review B* **1996**, 54, 11169.

[6] J. P. Perdew, K. Burke, M. Ernzerhof, Generalized gradient approximation made simple, *Physical review letters* **1996**, 77, 3865.

[7] a)J. Heyd, G. E. Scuseria, M. Ernzerhof, Hybrid functionals based on a screened Coulomb potential, *The Journal of chemical physics* **2003**, 118, 8207; b)A. V. Krukau, O. A. Vydrov, A. F. Izmaylov, G. E. Scuseria, Influence of the exchange screening parameter on the performance of screened hybrid functionals, *The Journal of chemical physics* **2006**, 125.

[8] M. Choi, F. Oba, Y. Kumagai, I. Tanaka, Anti-ferrodistortive-like oxygen-octahedron rotation induced by the oxygen vacancy in cubic SrTiO_3_, *Advanced Materials (Deerfield Beach, Fla.)* **2012**, 25, 86.

[9] H. J. Monkhorst, J. D. Pack, Special points for Brillouin-zone integrations, *Physical review B* **1976**, 13, 5188.

[10] A. T. Mulder, N. A. Benedek, J. M. Rondinelli, C. J. Fennie, Turning ABO_3_ antiferroelectrics into ferroelectrics: design rules for practical rotation‐driven ferroelectricity in double perovskites and A_3_B_2_O_7_ Ruddlesden‐Popper compounds, *Adv. Funct. Mater.* **2013**, 23, 4810.

[11] a)B. Su, M. Li, E. Song, Z. Xia, Sb^3+^‐Doping in Cesium Zinc Halides Single Crystals Enabling High‐Efficiency Near‐Infrared Emission, *Adv. Funct. Mater.* **2021**, 31, 2105316; b)X. Li, Y. Zheng, R. Ma, Z. Huang, C. Wang, M. Zhu, F. Jiang, Y. Du, X. Chen, B. Huang, F. Wang, B. Wang, Y. Wang, D. Peng, Broadband multimodal emission in Sb-doped CaZnOS-layered semiconductors, *Sci. China Mater.* **2021**, 65, 1329.

[12] K. Smith, S. Ramkumar, N. Harms, A. Clune, X. Xu, S.-W. Cheong, Z. Liu, E. Nowadnick, J. Musfeldt, Revealing pressure-driven structural transitions in the hybrid improper ferroelectric Sr_3_Sn_2_O_7_, *Physical Review B* **2021**, 104, 064106.

[13] X. Sun, Z. Tang, X. Yang, Z. Gao, Y. Wu, J. Jiang, Z. Zhang, S. Jiao, D. Li, H.-L. Cai, Structural evolution and phase transition of Sr_3_Sn_2_O_7_ doped with Ca, *Chem. Phys. Lett.* **2021**, 766, 138319.

[14] J. Lu, X. Liu, X. Ma, M. Fu, A. Yuan, Y. Wu, X. Chen, Crystal structures, dielectric properties, and phase transition in hybrid improper ferroelectric Sr_3_Sn_2_O_7_-based ceramics, *J. Appl. Phys.* **2019**, 125.

[15] a)A. Dobrowolska, A. J. Bos, P. Dorenbos, Electron tunnelling phenomena in YPO_4_: Ce, Ln (Ln= Er, Ho, Nd, Dy), *J. Phys. D: Appl. Phys.* **2014**, 47, 335301; b)P. Dorenbos, The hole picture as alternative for the common electron picture to describe hole trapping and luminescence quenching, *J. Lumin.* **2018**, 197, 62; c)P. Dorenbos, A. Bos, N. Poolton, Carrier recombination processes and divalent lanthanide spectroscopy in YPO_4_: Ce^3+^; L^3+^(L= Sm, Dy, Tm), *Physical Review B—Condensed Matter and Materials Physics* **2010**, 82, 195127.

[16] a)S. Dutta, S. Chattopadhyay, D. Jana, A. Banerjee, S. Manik, S. Pradhan, M. Sutradhar, A. Sarkar, Annealing effect on nano-ZnO powder studied from positron lifetime and optical absorption spectroscopy, *J. Appl. Phys.* **2006**, 100; b)M. Kong, Y. Li, X. Chen, T. Tian, P. Fang, F. Zheng, X. Zhao, Tuning the relative concentration ratio of bulk defects to surface defects in TiO_2_ nanocrystals leads to high photocatalytic efficiency, *J. Am. Chem. Soc.* **2011**, 133, 16414.

[17] a)M. Kostrzewa, A. S. S. Reddy, A. Ingram, A. Smirnov, V. R. Kumar, N. Veeraiah, Exploration of nano sized defects in Fe_2_O_3_ doped lead zirconium silicate glass ceramics by using positron annihilation lifetime spectroscopy, *Ceram. Int.* **2021**, 47, 21785; b)X. Liu, K. Zhou, L. Wang, B. Wang, Y. Li, Oxygen vacancy clusters promoting reducibility and activity of ceria nanorods, *Journal of the American Chemical Society* **2009**, 131, 3140.

[18] a)J.-J. Li, B. Weng, S.-C. Cai, J. Chen, H.-P. Jia, Y.-J. Xu, Efficient promotion of charge transfer and separation in hydrogenated TiO_2_/WO_3_ with rich surface-oxygen-vacancies for photodecomposition of gaseous toluene, *J. Hazard. Mater.* **2018**, 342, 661; b)Z. Wei, W. Wang, W. Li, X. Bai, J. Zhao, E. C. Tse, D. L. Phillips, Y. Zhu, Steering electron–hole migration pathways using oxygen vacancies in tungsten oxides to enhance their photocatalytic oxygen evolution performance, *Angew. Chem. Int. Ed.* **2021**, 60, 8236.

[19] a)V. P. Zakaznova-Herzog, S. Harmer, H. Nesbitt, G. Bancroft, R. Flemming, A. Pratt, High resolution XPS study of the large-band-gap semiconductor stibnite (Sb_2_S_3_): structural contributions and surface reconstruction, *Surf. Sci.* **2006**, 600, 348; b)T. Honma, R. Sato, Y. Benino, T. Komatsu, V. Dimitrov, Electronic polarizability, optical basicity and XPS spectra of Sb_2_O_3_–B_2_O_3_ glasses, *J. Non-Cryst. Solids* **2000**, 272, 1.
